# Coordinated Regulation of Intestinal Functions in *C. elegans* by LIN-35/Rb and SLR-2

**DOI:** 10.1371/journal.pgen.1000059

**Published:** 2008-04-25

**Authors:** Natalia V. Kirienko, John D. K. McEnerney, David S. Fay

**Affiliations:** Department of Molecular Biology, College of Agriculture, University of Wyoming, Laramie, Wyoming, United States of America; Stanford University Medical Center, United States of America

## Abstract

LIN-35 is the sole *C. elegans* representative of the pocket protein family, which includes the mammalian Retinoblastoma protein pRb and its paralogs p107 and p130. In addition to having a well-established and central role in cell cycle regulation, pocket proteins have been increasingly implicated in the control of critical and diverse developmental and cellular processes. To gain a greater understanding of the roles of pocket proteins during development, we have characterized a synthetic genetic interaction between *lin-35* and *slr-2*, which we show encodes a C2H2-type Zn-finger protein. Whereas animals harboring single mutations in *lin-35* or *slr-2* are viable and fertile, *lin-35*; *slr-2* double mutants arrest uniformly in early larval development without obvious morphological defects. Using a combination of approaches including transcriptome profiling, mosaic analysis, starvation assays, and expression analysis, we demonstrate that both LIN-35 and SLR-2 act in the intestine to regulate the expression of many genes required for normal nutrient utilization. These findings represent a novel role for pRb family members in the maintenance of organ function. Our studies also shed light on the mechanistic basis of genetic redundancy among transcriptional regulators and suggest that synthetic interactions may result from the synergistic misregulation of one or more common targets.

## Author Summary

Genetic or functional redundancy is a widespread feature of eukaryotic genomes and may be largely attributable to the complex and overlapping nature of many regulatory networks. Despite the prevalence and importance of this phenomenon, the mechanistic bases underlying genetic redundancy have remained elusive, particularly within the context of multicellular developing organisms. To gain a deeper understanding of this phenomenon, we have focused on a synthetic genetic interaction between *lin-35*, a *C. elegans* member of the pRb/pocket-protein tumor suppressor family and *slr-2*, a Zn-finger protein. Whereas single mutants in either *lin-35* or *slr-2* develop normally, *lin-35*; *slr-2* double mutants display a highly penetrant synthetic growth arrest during early larval development. We show that this arrest is specifically due to an inability to utilize standard nutrient sources. Using a wide range of approaches including transcriptome profiling, mosaic analysis, and expression analysis, we demonstrate that both LIN-35 and SLR-2 act within the intestine to regulate the expression of many genes that function in nutrient utilization. We also show that the basis of the synthetic interaction between *lin-35* and *slr-2* is not merely due to regulon overlap, but likely results from the synergistic misregulation of multiple shared transcriptional targets in the intestine.

## Introduction

The Retinoblastoma protein, pRb, was among the first recognized tumor suppressor proteins [1]–[3], and loss or repression of pRb function is thought to play a causative role in most human cancers [4]–[8]. The role of pRb as a tumor suppressor has been largely attributed to its functions in cell cycle regulation, which it carries out in conjunction with its two family members, p107 and p130, collectively known as the pocket proteins [9]–[11]. Pocket proteins act primarily as transcriptional repressors and physically associate with diverse array of transcription factors [12]. The most thoroughly characterized of these interactions is with E2F family members, which leads to the repression of E2F-target genes, a group that includes many genes required for entry and progression through S-phase [13]–[16]. Correspondingly, LIN-35, the sole pocket protein ortholog in *C. elegans*, carries out analogous cell cycle functions during larval stages of development [17]–[21].

In addition, a growing number of studies have demonstrated non−cell cycle roles for pRb family members, which in some cases may prove relevant to the tumor-suppressing activity of pocket proteins [12],[19],[22],[23]. In the case of LIN-35, the majority of these functions are revealed only when LIN-35 activity is compromised in specific mutant backgrounds. This phenomenon can be explained on the basis of genetic or functional redundancy, a widespread feature of eukaryotic genomes, which is attributable to the complex and overlapping nature of many regulatory networks. The first described, and still most thoroughly characterized, genetically redundant function of LIN-35 is its role restricting epidermal cells from inappropriately acquiring vulval cell fates [22],[24],[25]. More specifically, when *lin-35*, a member of the class B group of synthetic multivulval (SynMuv) genes, is simultaneously inactivated with individual members of the SynMuv A or C classes [24],[26], hyperinduction of vulval cells is observed. In contrast, single mutants in most SynMuv genes, including *lin-35*, do not display observable defects in vulval development.

LIN-35 also redundantly regulates pharyngeal and vulval morphogenesis [27]–[29], asymmetric cell divisions [30], cell fates in the somatic gonad [31], larval growth and development [30],[32],[33], and the promotion of cell death [34]. Furthermore, *lin-35* functions non-redundantly in the control of germline gene repression [35] and germline apoptosis [36] and to modulate sensitivity to RNAi [35],[37]. In addition, transcriptome profiling has suggested potential roles for LIN-35 in intestinal and neuronal development, although direct evidence for functions in these tissues has been lacking [21]. Here we describe a novel role for LIN-35 in the intestine of *C. elegans*. Specifically, we find that LIN-35, in conjunction with the Zn-finger protein SLR-2, acts within intestinal cells to regulate the expression of genes required for proper nutrient utilization.

## Results

### *lin-35/Rb* and *slr-2* Are Genetically Redundant

**Figure 1:**
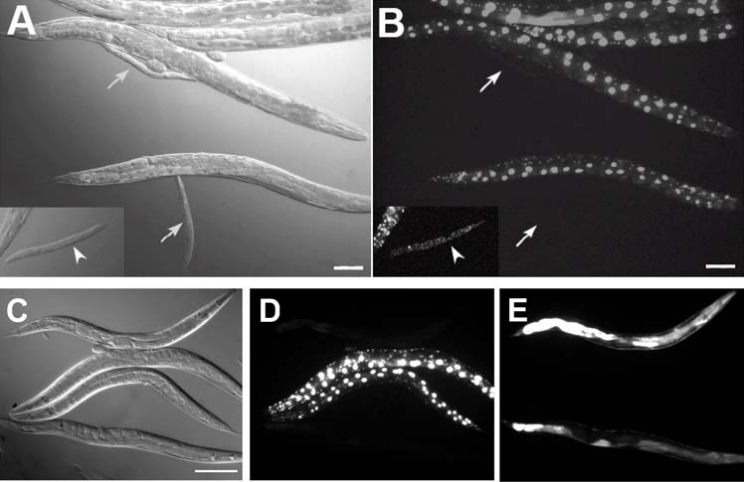
*lin-35* and *slr-2* are genetically redundant. (A, B) DIC (A) and corresponding GFP fluorescence (B) images of *lin-35*(*n745*); *slr-2*(*ku297*) hermaphrodites. The large adult GFP^+^ animals carry the *kuEx119* extrachromosomal array, which expresses wild-type *lin-35* along with the *sur-5*::GFP marker. Arrows indicate sibling progeny that failed to inherit the *kuEx119* array and are arrested in early larval development. The inset (A, B) shows a larval-arrested *lin-35*; *slr-2*; *kuEx119* double mutant following *lin-35(RNAi)* treatment. (C−E) DIC (C) and corresponding GFP (D) and RFP (E) fluorescence images of *lin-35*(*n745*); *slr-2*(*ku297*) adult hermaphrodites carrying either the *kuEx119 lin-35* (D) or CBG05648–*sur-5*::RFP (E) rescuing arrays. Note that whereas *sur-5*::GFP is nuclear, the *sur-5*::RFP marker is cytosolic and nuclear. CBG05648 is the *C. briggsae* ortholog of *slr-2*/Y59A8B.13. Scale bars: in A, B 100 µm; in C, 200 µm for C–E.

A previously described genetic screen was used to identify genes that function redundantly with *lin-35* [20]. Briefly, we chemically mutagenized *lin-35*(*n745*) mutants that carry an unstable extrachromosomal array (*kuEx119*), which expresses wild-type *lin-35* together with the *sur-5*::GFP marker. Following F2 clonal selection, we identified strains with synthetic interactions by the presence of visible phenotypes in progeny that failed to inherit the array. One allele, *ku297*, defines a locus that we have designated as *slr-2* (for *synthetic with lin-35/Rb*). *slr-2* single mutants are largely indistinguishable from wild type, although we observed weak-to-moderate elongation defects at low frequencies (the Dpy phenotype). In contrast, *lin-35*; *slr-2* double mutants exhibit uniform early-larval arrest (Figure 1A, 1B, Table 1). To verify that phenotypic alleviation by *kuEx119* was specifically due to rescue of *lin-35* activity, we used RNAi to knock down the expression of *lin-35* from the array. This resulted in animals that arrested in early larval development despite the presence of the array (Figure 1A, 1B, inset), confirming that the interaction was specific to *lin-35* and *slr-2*.

**Table 1:** *slr-2* interactions with SynMuv genes

Strain	%Larval arrest (n)
*slr-2*	1 (360)
*lin-35*	1 (213)
*lin-35;slr-2*	100 (1000)
*dpl-1*	7 (181)
*dpl-1;slr-2*	83 (798)
*hpl-2*	0 (118)
*hpl-2;slr-2*	2 (444)
*lin-9*	2 (101)
*lin-9;slr-2*	7 (308)
*lin-15b*	0 (184)
*lin-15b;slr-2*	0 (187)
*lin-36*	0 (154)
*lin-36;slr-2*	3 (165)
*lin-37*	1 (228)
*lin-37;slr-2*	5 (319)
*lin-53*	0 (120)
*lin-53;slr-2*	3 (179)
*lin-15a*	0 (170)
*lin-15a;slr-2*	1 (250)

For information on specific alleles, see Materials and Methods.

### *slr-2* Encodes the C2H2 Zinc Finger Y59A8B.13

**Figure 2:**
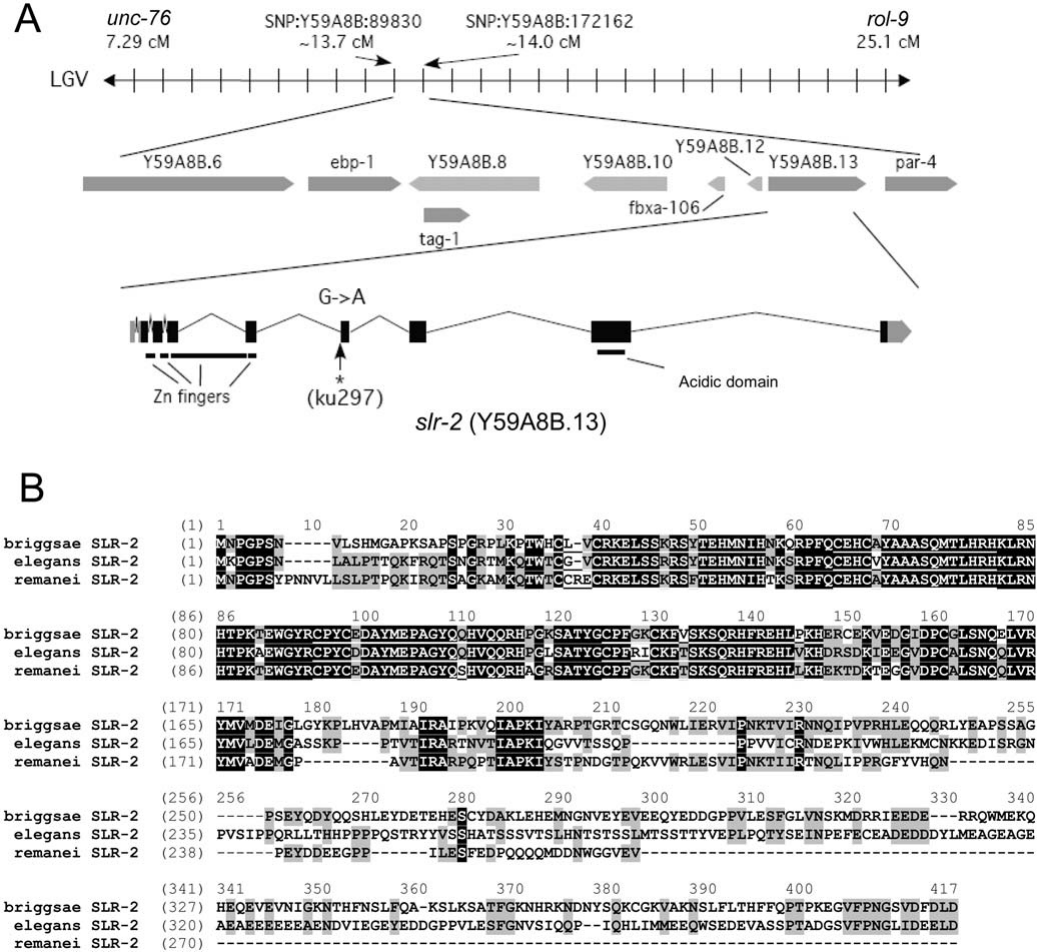
Identification of the *slr-2* locus. (A) The 82.3-kb genomic region that encompasses the area defined by SNP mapping to harbor the *slr-2* locus, along with the verified gene structure of *slr-2*, based on the analysis of *slr-2* cDNAs. Also indicated are the locations of the Zn-finger and acidic domains and the molecular lesion identified in *ku297* mutants. The lesion alters the conserved splice acceptor site preceding exon six and effectively abolishes splicing between exons five and six. (B) An alignment of the predicted *C. elegans* SLR-2 peptide (based on the *slr-2* cDNA), together with its putative orthologs in *C. briggsae* (CBG05648) and *C. remanei*. The arrow indicates the location of the frameshift in *ku297* mutants.

We mapped *ku297* to an ∼82-kb region of LGV that contains nine genes, including a predicted C2H2-type Zn-finger protein, Y59A8B.13 (Figure 2A). Given the established role of *lin-35*/Rb in transcriptional regulation as well as our previous findings that *lin-35* displays synthetic genetic interactions with other transcriptional regulators, we focused on Y59A8B.13 as a candidate locus. The large size of the Y59A8B.13 genomic locus along with the presence of multiple repetitive elements within this region precluded our amplification and cloning of the Y59A8B.13 locus from *C. elegans*. We therefore turned to the Y59A8B.13 ortholog from *C. briggsae*, CBG05648, which though strongly conserved at the amino acid level with Y59A8B.13, comprises a smaller and non-repetitive genomic region (Figure 2B). Injection of a PCR product spanning the complete predicted CBG05648 locus together with a *sur-5*::RFP plasmid into *lin-35*; *slr-2*; *kuEx119* hermaphrodites led to the generation of RFP-marked extrachromosomal arrays in seven independent strains. Strikingly, all seven strains were strongly rescued for the *lin-35*; *slr-2* larval-arrest phenotype by arrays containing the *C. briggsae* Y59A8B.13 ortholog (Figure 1C–E). This result is consistent with previous findings demonstrating the ability of *C. briggsae* genes to rescue corresponding mutants in *C. elegans* [38]. Based on these findings, as well as additional data presented below, we conclude that Y59A8B.13 is SLR-2. Our results also underscore the utility of trans-species rescue approaches in cases where technical limitations may preclude the use of the endogenous locus.

Using a combination of approaches, we identified a full-length cDNA corresponding to *slr-2* that was trans-spliced at the 5′ end to the SL1 spliced leader sequence (Figure S1) [39]. We note that our cDNA-derived sequence for *slr-2* differed from WormBase predictions in the location of several exons. That the translation of *slr-2* is likely to proceed from the second 5′ ATG is suggested by sequence alignments with the closely related *C. briggsae* and *C. remanei* orthologs (Figure 2B). All three proteins show strong sequence identity within the N-terminal Zn-finger regions but are divergent in their C-terminal domains (Figure 2B). The translation of *slr-2* also predicts what appears to be an acidic domain in the C terminus, consistent with SLR-2 functioning as a transcriptional regulator.

Sequencing of the entire *slr-2* genomic region in *ku297* mutants revealed a single mutation within the splice acceptor site preceding exon six (Figure 2A) [40]. This mutation, which affects the terminal invariant nucleotide, would be predicted to strongly disrupt splicing between exons five and six (e5–e6), leading to aberrant splicing between exons five and seven (e5–e7). The resultant transcript would contain a frameshift followed by a premature stop codon at the eleventh nucleotide of exon 7, leading to the deletion of the C-terminal 250 amino acids of SLR-2. We confirmed this prediction by quantitative real-time PCR analysis, in which we observed a ∼1000-fold decrease in transcript abundance of the e5–e6 product versus the e4–e5 control (data not shown). Furthermore, we observed an e5–e7 splicing product that was ∼80 bp shorter than wild type, consistent with the absence of the 83-bp sixth exon (data not shown). Taken together, these data demonstrate that processing of the normal *slr-2* transcript is dramatically reduced in *ku297* mutants, suggesting that *ku297* likely represents a strong loss-of-function or null allele. Consistent with this interpretation, when placed over a regional deficiency that removes the entire *slr-2* locus, *slr-2/yDf4*, transheterozygotes displayed no exacerbation of the weak Dpy phenotype associated with *slr-2* single mutants and were viable and fertile.

### Transcriptome Analysis of *slr-2* Mutants Reveals Functions Associated with the Intestine

As our analysis of arrested *lin-35*; *slr-2* larvae failed to reveal any obvious morphological defects, we undertook transcriptome profiling as a means for shedding light the basis of the double-mutant phenotype. Our rationale for this approach stemmed in part from the known roles of LIN-35 and pRb family members in transcriptional control, as well as the presence of four Zn fingers and an acidic domain in SLR-2, which strongly suggest that it too may function as a transcriptional regulator. Thus, we reasoned that the observed genetic redundancy could be due to the misregulation of targets that are common to both regulators. More specifically, we had previously observed two major classes of genes affected in *lin-35* mutants at larval stages: cell cycle control and intestinally expressed genes [21]. We therefore hypothesized that SLR-2 may co-regulate genes in either or both of these classes.

Because *lin-35*; *slr-2* mutants arrest in early larval development, we focused on the late L1 stage for our microarray analysis. Transcriptome profiling was carried out on three independent biological replicates using Affymetrix GeneChips and established procedures [21]. Our analysis identified ∼1,700 genes that are differentially regulated in *slr-2*(*ku297*) mutants as compared with identically staged wild-type animals (Figure S2). We further verified expression changes for 29 of these targets by qRT-PCR, thus validating findings from the microarray data (Figure S3 and Table S1).

**Figure 3:**
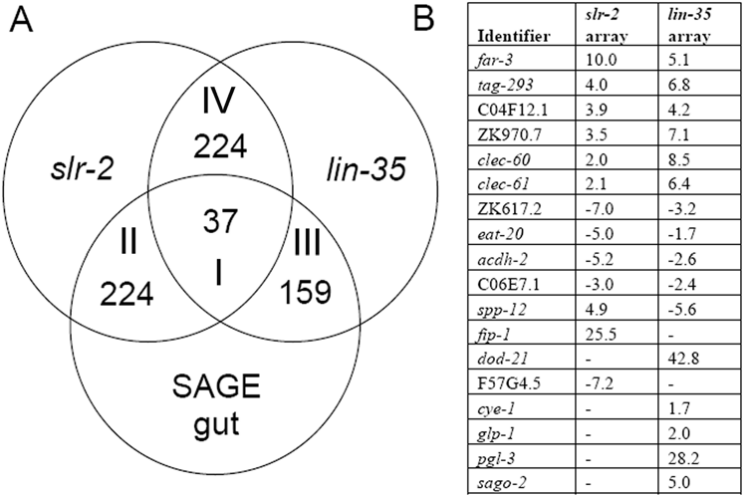
Comparative transcriptome profiles of *lin-35* and *slr-2* mutants with SAGE-derived intestinal-specific genes. (A) The overlap in genes identified by the three independent analyses (also see text). Note the large overlap in genes misregulated in both *lin-35* and *slr-2* mutants (p<0.001), as well as the strong correlation with both mutants and intestinal-specific genes (p<0.001) [21],[41]. (B) Corresponding qRT-PCR data for a selected subset of *lin-35*- and *slr-2*-regulated genes. Note the lack of correlation between *lin-35* and *slr-2* mutants in the misregulation of cell cycle (*cye-1*), germline (*pgl-3* and *glp-1*), and RNAi-associated (*sago-2*) genes. Other genes listed have functions ascribed to the intestine and metabolism (also see text and Table S1).

In common with our previous findings for *lin-35*, the *slr-2* data set showed strong overrepresentation of intestine-enriched/intestine-specific genes (p<0.001) as previously identified using a serial analysis of gene expression (SAGE) approach [41]; 261 genes were common to both data sets (Figure 3A, categories I and II; Figure S4, also see Materials and Methods). A comparison of differentially regulated genes in *slr-2* and *lin-35* single mutants also revealed a statistically significant overlap (p<0.001); 261 genes were present in both data sets (Figure 3, categories I and IV; Figure S5). Furthermore, although *lin-35* and *slr-2*-responsive genes showed opposite trends in their directionality of regulation (70% of *lin-35* targets were upregulated versus 20% of *slr-2* targets), the correlation coefficient calculated for common targets was 0.58, indicating a moderate-to-strong correlation. In addition, 76% of genes common to both data sets showed expression changes in the same direction; the common data set contained an approximately equal mixture of up- and downregulated genes.

Importantly, *lin-35* and *slr-2*-responsive genes displaying overlap with the SAGE dataset (Figure 3, categories I, II, and III) also showed evidence of intestinal gene enrichment and intestine-associated functions according to several additional lines of evidence. Based on meta-array functional clustering [42], the only mountains showing strong overrepresentation (p<0.001) were those associated with the intestine, amino acid metabolism, and lipid metabolism (Figure S6A). In addition, available data from the *C. elegans* expression database (http://gfpweb.aecom.yu.edu), showed that, on average, 78% of genes were expressed in the intestine and 38% showed intestine-specific expression (Figure S6B). Similar results were also obtained from an examination of in situ hybridization data available on the NEXTDB database (http://nematode.lab.nig.as.jp/db2/index.php) (Figure S6C). Moreover, genes found to overlap between *lin-35* and *slr-2* only (Figure 3, category IV), also showed a specific overrepresentation of intestine and metabolic mountains (p<0.001), a finding further corroborated by data available through the expression databases (Figure S6A-C). Thus, genes implicated in intestinal and metabolic functions are statistically and uniquely overrepresented among the common targets of *lin-35* and *slr-2*, including many genes not previously identified by SAGE analysis.

A previous analysis of intestine-specific/enriched genes identified a single over-represented motif (TGATAA), corresponding to the binding site of the intestinal regulator, ELT-2 [41]. This motif is present in the proximal enhancer regions of 23.3% of genes (using multiple random sampling) in the intestine-specific/enriched dataset used in our above analysis. Using cluster analysis, we independently identified this motif among *lin-35* responsive genes [21], and have observed a high frequency of this motif (33–62%) in categories I–IV of overlapping genes from our current analysis (Figure 3A and Figure S6D). This finding is further consistent with our above analysis, indicating that intestine-associated genes our enriched in our dataset. Consistent with the misregulation of intestinal genes, *slr-2* mutants exhibited repression of several important metabolic pathways, including the TOR and insulin signaling networks (Table S1), suggesting that *slr-2*(*ku297*) mutants experience metabolic stress [43].

In contrast to intestinal genes, genes with cell cycle functions were not overrepresented in the *slr-2* data set (Figure S3). Therefore, the genetic redundancy observed for *lin-35* and *slr-2* mutants is consistent with their combined effects on intestinal gene misregulation and not to cell cycle defects (also see below). We also note that in addition to intestinal genes, the *lin-35* and *slr-2* data sets showed upregulation of ∼15 common genes with attributed neurological functions. Based on data presented below, it is unlikely, however, that the misregulation of these genes contributes strongly to the double-mutant phenotype. Finally, we observed the downregulation of several *dpy* genes in *slr-2* mutants (*dpy-11*, *dpy-18*, and *dpy-21*), which may account for the variable morphogenetic defects observed in these animals.

### *lin-35* and *slr-2* Carry Out Redundant Functions within the Intestine

The larval-arrest phenotype of *lin-35*; *slr-2* mutants, along with the results of our transcriptome analysis, are consistent with LIN-35 and SLR-2 acting within the intestine to control the expression of gut-associated genes. To determine the precise tissue focus for LIN-35 and SLR-2, we carried out a mosaic analysis [44]–[47]. This method takes advantage of the inherent mitotic instability of most extrachromosomal arrays and allows for the identification of particular mosaic species, thereby enabling direct correlations to be drawn between gene function and localized expression.

**Figure 4:**
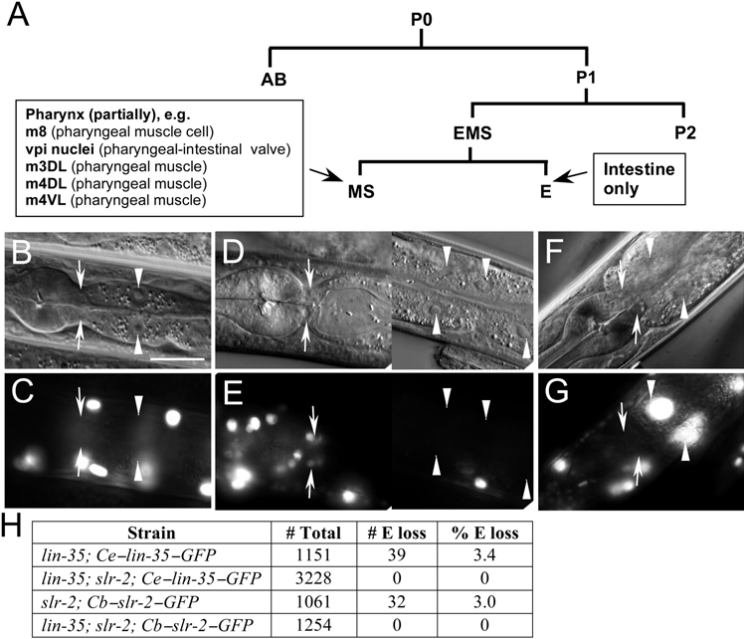
*lin-35* and *slr-2* act in the intestine. (A) Relevant cells and their progenitors from the embryonic lineage used in the mosaic analysis. (B−G) DIC (B, D, F) and corresponding GFP fluorescence (C, E ,G) images of *lin-35*; *slr-2*; *kuEx119* young adults. Arrows indicate the locations of the MS-derived vpi cells; arrowheads, intestinal nuclei. (B, C) A representative EMS-mosaic animal where the *kuEx119* array failed to be segregated to EMS. Note the absence of GFP expression in E-derived intestinal cells as well as in MS-derived vpi cells and MS-derived cells of the posterior pharyngeal bulb (left-most circular structure). (D, E) An E-mosaic animal. Note the presence of GFP expression in vpi cells as well as in posterior pharyngeal cells (left). (F, G) An MS-mosaic animal. Note the presence of GFP expression in intestinal cells along with a lack of GFP expression in vpi and posterior-most pharyngeal cells. (H) The frequency of array loss within the intestine for the *C. elegans lin-35*–GFP (*kuEx119*) and *C. briggsae slr-2*–GFP (*fdEx25*) arrays in *lin-35* and *slr-2* single mutants, respectively. In contrast, note the complete absence of intestinal-mosaic animals for the same arrays in *lin-35*; *slr-2* double mutants. Scale bars: in B, 10 µm for B–G.

To assess the roles of *lin-35* and *slr-2* in the intestine, we first measured the frequencies by which the *C. elegans lin-35* (*Ce–lin-35*; *kuEx119*) and *C. briggsae slr-2* (*Cb–slr-2*; *fdEx25*) rescuing extrachromosomal arrays are spontaneously lost within the intestinal lineage in *lin-35* and *slr-2* single mutants, respectively. Under these non-selective conditions, intestinal loss was observed in 3.4% and 3.0% of adults carrying the *Ce–lin-35* and *Cb–slr-2* arrays, respectively (Figure 4H). In contrast, loss of the *Ce–lin-35* array in intestinal cells was never observed in adult *lin-35*; *slr-2* double mutants, suggesting that expression of *lin-35* from the array is required within intestinal cells for rescue of larval arrest (Figure 4H). Similar results were obtained for the *Cb–slr-2* array, indicating that *slr-2* also acts within the intestine (Figure 4H).

One limitation to the above analysis is that the absence of intestinal expression in mosaic animals could reflect loss of the array in either the E blastomere (Figure 4D, 4E), which gives rise exclusively to intestinal cells, or in EMS (Figure 4B, 4C) or P1, which are progenitors of E but produce additional cell types (Figure 4A). Thus we determined the frequency of E-specific losses by examining array expression in additional relevant lineages of intestinal mosaic animals. E-specific losses were found to account for 36% of *Ce–lin-35* intestinal mosaic animals (n = 11), which is close to the expected frequency based on the lineage (i.e., the failure to segregate the array during one of three possible cell divisions or 33%). Thus of the total number of *kuEx119* intestinal mosaic animals (3.4%), we would expect that 36% had experienced loss specifically within the E-cell lineage. This is equivalent to ∼1.2% (or 0.034×0.36) of all animals carrying the *Ce–lin-35* array. Thus, if *lin-35* is not required within the E-lineage, we would expect to observe ∼40 viable intestinal-mosaic adults among the 3,228 *lin-35*; *slr-2* animals assayed. The total absence of intestinal-mosaic adults strongly indicates that *lin-35* function is indeed required in the intestine for rescue of *lin-35*; *slr-2* mutants. Correspondingly, E-specific losses accounted for 40% of *Cb−slr-2* intestinal mosaic animals (n = 12). Thus, among the 1,254 *lin-35*; *slr-2*; *fdEx25* animals assayed, ∼15 E-specific mosaic animals would have been expected. As for *lin-35*, the complete absence of intestinal mosaics in the adult population demonstrates that *slr-2* activity is also required in the intestine (Figure 4H).

The above results conclusively demonstrate that *lin-35* and *slr-2* are required in the E-cell lineage for rescue of double-mutant lethality. However, these findings do not rule out the possibility that these genes may be simultaneously required in another lineages, such as MS and AB, which are essential for formation of the foregut (Figure 4A). To address the role of MS, we specifically screened for mosaic double-mutant animals in which the *Ce–lin-35* array was absent from the MS lineage but was present in E. In doing so, we identified five *lin-35*; *slr-2*; *kuEx119* viable adults in which the array was absent from the entire MS lineage (Figure 4F, 4G). In addition, we identified a number of rescued adults in which the *Ce–lin-35* array was missing from within sub-lineages of MS. Given that viable adults were identified that lacked both *lin-35* and *slr-2* within the MS lineage, these data demonstrate that neither *lin-35* nor *slr-2* are required within MS for rescue of *lin-35*; *slr-2* larval lethality. Similarly, we identified viable double-mutant adults where expression patterns indicted the absence of the *Ce–lin-35* array within the AB.a (n = 4) and AB.p (n = 3) lineages, suggesting that neither *lin-35* nor *slr-2* activity are required within the AB lineage for the rescue of double mutants. Taken together, these results indicate that *lin-35* activity is not required in the foregut (or other lineages produced by AB and MS) for rescue of *lin-35*; *slr-2* double-mutants.

Previously, we and others have observed a redundant role for *lin-35* in the control of intestinal cell proliferation [17],[20]. Given the above indicated intestinal focus of the *lin-35*; *slr-2* phenotype, we examined staged double mutants for abnormalities in intestinal nuclei number and DNA ploidy. We observed wild-type numbers of intestinal nuclei in 54% of *lin-35*; *slr-2* mutants (20±1), although 46% contained 1–14 extra nuclei (Figure S7). This result demonstrates that *lin-35*; *slr-2* mutants undergo larval arrest despite, in most cases, having normal numbers of intestinal nuclei. As a control, we also examined intestinal nuclei numbers in larvae containing a loss-of-function mutation in the APC component, *fzr-1*, and observed 1–14 extra nuclei in 72% of these animals (Figure S7). Given that only 2% of *fzr-1* mutants undergo arrest during larval development (n = 237), this result demonstrates that extra intestinal nuclei per se do not cause larval arrest, a result that is consistent with previous reports [17],[20]. We also failed to detect any difference in DNA ploidy between intestinal cells in wild type and *lin-35*; *slr-2* mutants (Figure S7C-D). Thus, growth arrest in *lin-35*; *slr-2* mutants due to intestinal-associated defects cannot be attributed to overt cell cycle abnormalities.

### *lin-35*; *slr-2* Mutants Are Defective at Nutrient Utilization

**Figure 5:**
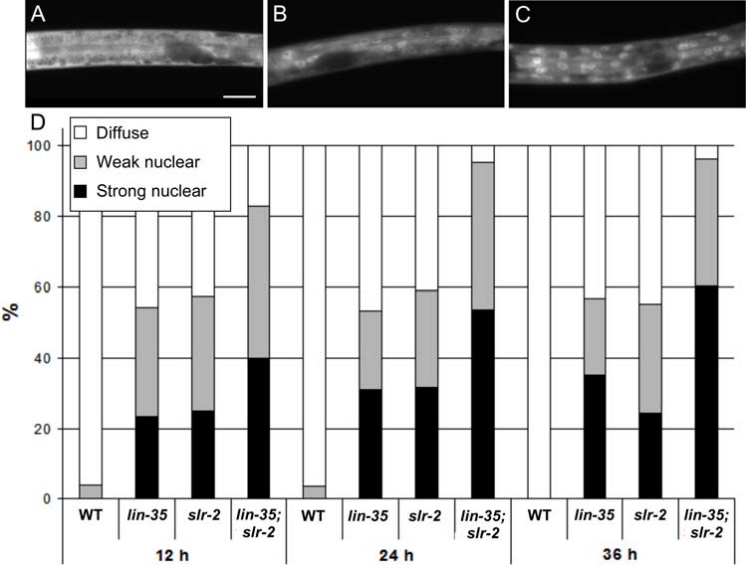
DAF-16::GFP localization in *lin-35*; *slr-2* mutants. (A−C) GFP fluorescence representing diffuse (A), weak-nuclear (B), and strong-nuclear (C) localization of the DAF-16::GFP marker. Genotypes of depicted L1 larvae are wild type (A), *slr-2*, (B) and *lin-35*; *slr-2* (C). (D) Relative rates of the three classes of GFP localization for wild-type (N2), *lin-35* and *slr-2* single mutants, and *lin-35*; *slr-2* double mutants at 12, 24, and 36 hours after synchronization onto an abundant bacterial food source (OP50). *lin-35*; *slr-2* double mutants show high levels of DAF-16::GFP nuclear localization by 12 hours, consistent with starvation. Also note that both *lin-35* and *slr-2* single mutants, which are not growth arrested but show significant misregulation of intestinal genes, exhibit significantly higher levels of DAF-16::GFP than wild type. Scale bars: in A, 10 µm for A–C.

The above findings indicate that *lin-35*; *slr-2* double mutants are likely to undergo early larval arrest as a result of intestinal-specific gene misregulation. More precisely, malfunctioning of the intestine in double mutants may lead to nutrient deprivation and subsequent arrested growth. To determine whether *lin-35*; *slr-2* animals are defective at nutrient utilization, we made use of the DAF-16::GFP translational fusion reporter. DAF-16::GFP exhibits diffuse cytoplasmic expression throughout the body in animals that are well fed but rapidly translocates to nuclei following their removal from a food source [48]. Accordingly, in wild type we observed diffuse GFP expression in fed animals at all stages and nuclear localization in populations after nutrient deprivation (Figure 5A, 5D, and data not shown). In contrast, *lin-35*; *slr-2* double-mutant larvae displayed high levels of nuclear DAF-16::GFP expression on plates with ample food as early as 12 hours into larval development (Figure 5C, 5D). Nuclear GFP localization further increased in double mutants at later time points, such that nearly 100% of animals displayed punctate fluorescence by 36 hours (Figure 5D). This result suggests that *lin-35*; *slr-2* larvae experience starvation in the presence of a food source, consistent with defects in intestinal functions. We also note that as compared with wild type, both *lin-35* and *slr-2* single mutants exhibited enhanced nuclear DAF-16::GFP localization on plates containing food at all time points (Figure 5B, 5D). This result is perhaps not unexpected given that both single mutants exhibit mild growth retardation (data not shown) and independently show misregulation in many intestine-related genes.

**Figure 6:**
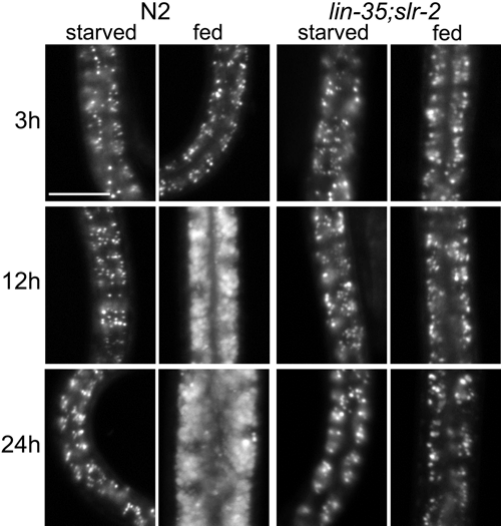
UV intestinal autofluorescence in *lin-35*; *slr-2* mutants. Representative images from a time course following placement of synchronized wild type and *lin-35*; *slr-2* L1 larvae on plates devoid of a bacterial food source (starved) or containing OP50 *E. coli* (fed). Intestinal autofluorescence in wild-type starved animals is much weaker than in fed animals and appears punctate. Note that both starved and fed *lin-35*; *slr-2* mutants appear identical to each other and to starved wild-type animals at the 12 h and 24 h time points. For quantitation of the presented data and information on single mutants, see Figure S8. All images were captured using identical exposure times. Scale bar: in A, 10 µm for all panels.

Because nuclear localization of DAF-16::GFP is known to occur in response to several other forms of environmental stress [48], we performed an independent assay to test whether or not *lin-35*; *slr-2* mutants specifically experience nutritional deprivation. Previous studies have demonstrated that intestinal cell UV-induced autofluorescence, which facilitates the visualization of lysosomal gut granules that serve as sites of fat storage [49], provides a reliable marker for starvation in eating-defective mutants [50]. We therefore examined gut granule autofluorescence in wild-type, *lin-35*, *slr-2*, and *lin-35*; *slr-2* staged larvae propagated in the presence or absence of the OP50 food source. Strikingly, by this assay *lin-35*; *slr-2* mutants grown in the presence of food displayed a punctate pattern of gut autofluorescence that was identical to wild-type animals propagated in the absence of the food (Figure 6). In contrast, both single mutants were effectively indistinguishable from wild type under all conditions and time points tested Figure S8). Thus, by two independent assays, *lin-35*; *slr-2* mutants show evidence of experiencing nutritional deprivation.

Based on our evidence that *lin-35*; *slr-2* larvae have intestinal-associated defects and undergo starvation in the presence of their normal bacterial food source, *E. coli* (OP50), we hypothesized that suppression of larval arrest might be achievable by supplying double mutants with a preprocessed synthetic food source. To test this, we grew *lin-35*; *slr-2* animals in liquid axenic medium, a nutreint source consisting of peptides, fatty acids, hydrolyzed yeast and soy, dried milk, and hemoglobin [51]. Notably, 19% of double mutants grown in axenic medium were capable of bypassing early larval arrest and 5% of the total population went on to become gravid adults (n = 243). Furthermore, when fertile *lin-35*; *slr-2* adults were placed back onto OP50 plates, progeny from these animals arrested uniformly as L1 larvae. We note that although the observed frequency of suppression of larval arrest was relatively modest in these experiments, the ability of the synthetic medium to bypass what is otherwise a completely penetrant phenotype is highly significant. In addition, we examined the expression pattern of DAF-16::GFP in *lin-35*; *slr-2* mutants rescued from larval arrest by growth on axenic media. Notably, we observed cytosol-specific expression in 95% of rescued double-mutant adults (n = 21; Figure S9). This later finding indicates that the nuclear localization of DAF-16::GFP observed in previous experiments (Figure 5) occurs most likely as a consequence of nutritional deprivation and is not due to non-specific effects conferred by the *lin-35* and *slr-2* mutations.

It has previously been shown that developmental arrest resulting from nutrient deprivation is mediated during the L1 stage by a pathway that includes the DAF-18/PTEN lipid phosphatase [52]. One hallmark of this developmental arrest is the cessation of all germ cell proliferation, leading to diminutive gonads. Consistent with *lin-35*; *slr-2* mutants undergoing starvation-induced arrest, gonad size in double mutants was indistinguishable from wild-type starved L1s (Figure S10E). To determine whether or not germline proliferation is inhibited in *lin-35*; *slr-2* mutants by the DAF-18 pathway, we used RNAi feeding to inhibit *daf-18* activity in double mutants (See Materials and Methods). Most notably, average gonad size increased by 2.1-fold in double mutants exposed to *daf-18(RNAi)* (n = 55) versus OP50 controls (n = 59; Figure S10). This result is consistent with our findings indicating that growth arrest in double mutants is due to nutritional deprivation and further demonstrates that the developmental arrest exhibited by *lin-35*; *slr-2* mutants depends, at least in part, on the DAF-18 pathway.

Although data presented above, including the mosaic and transcriptome analyses, strongly implicates the intestine as the focus of the *lin-35*; *slr-2* growth-arrested phenotype, defects in bacterial cell uptake and mechanical disruption by the foregut (pharynx) could theoretically account for the starvation-induced arrest. To test for this possibility, we directly assayed the ability of *lin-35*; *slr-2* larvae to ingest a food analog (fluorescent beads) and observed normal uptake in 90% of double mutants at all time points tested (Figure S11). Furthermore, based on a GFP-marked OP50 strain, mechanical disruption of bacteria was completely normal in double mutants twelve hours into larval development, at which time double mutants show strong evidence of starvation-induced growth arrest (Figure S12; Figures 5 and 6). Taken together, our cumulative results strongly indicate that *lin-35*; *slr-2* double mutants undergo starvation-induced growth arrest and that this arrest is specifically attributable to defects associated with the intestine.

### *slr-2* Is Expressed in the Intestine and Foregut of Developing Larvae

**Figure 7:**
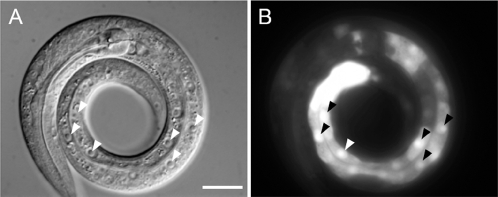
*slr-2* is expressed in the intestine. DIC (A) and corresponding GFP fluorescence (B) images. Approximately 900 bp of regulatory sequences upstream to the predicted *slr-2* start codon was used to drive expression of an integrated GFP reporter. Reporter expression was observed throughout the intestine in L1 larvae with highest levels consistently occurring in the posterior-most cells. Arrowheads indicate intestinal nuclei in both panels. Expression at additional stages can be seen in Figure S13. Scale bars: in A, 10 µm for A and B.

Our results indicate that both LIN-35 and SLR-2 function redundantly within the intestine to promote nutrient utilization. Consistent with this, LIN-35 is expressed in many cell types throughout early development including cells of the intestine [25],[53]. To determine the pattern of *slr-2* expression during development, we constructed a *slr-2*::GFP transcriptional reporter using an ∼900-bp region upstream of the *slr-2* start codon. Based on the location of a nearby adjacent gene, Y59A8B.12 (Figure 2A), this sequence is likely to encompass the complete 5′ regulatory region of *slr-2*. *slr-2*::GFP expression was first detected in most or all embryonic cells beginning at around the 100-cell stage (Figure S13). Beginning in late embryonic development and continuing through L1, *slr-2*::GFP expression was largely restricted to the intestine (Figure 7A, 7B; Figure S13), with highest levels of expression observed in posterior gut cells. A similar expression pattern was also observed in L4 larvae and adults, although expression was detected in additional tissues including the foregut (Figure S13). This expression pattern is consistent with our findings that *slr-2* acts during the L1 stage to control the expression of many genes associated with intestinal functions.

### *slr-2* Interactions with SynMuv Genes

As described in the Introduction, *lin-35* limits vulval cell induction in cooperation with numerous genes of the SynMuv network. To determine whether or not the observed genetic interaction with *slr-2* is specific to *lin-35* or is common among other SynMuv family members, we tested seven Class B and one Class A mutant for genetic interactions with *slr-2*. Interestingly, only one of the tested SynMuv genes, *dpl-1*, showed strong interactions with *slr-2* (Table 1). *dpl-1* encodes an ortholog of mammalian DP, which functions as a binding partner for the E2F family of transcriptional regulators. This finding therefore implicates DP and E2F as co-partners of LIN-35 in the transcriptional regulation of intestinal-associated genes. In contrast, the remaining mutants showed at most very weak interactions with *slr-2*, a finding that underscores the fundamental differences between the role of LIN-35 in vulval cell induction and nutrient utilization (Table 1).

**Figure 8:**
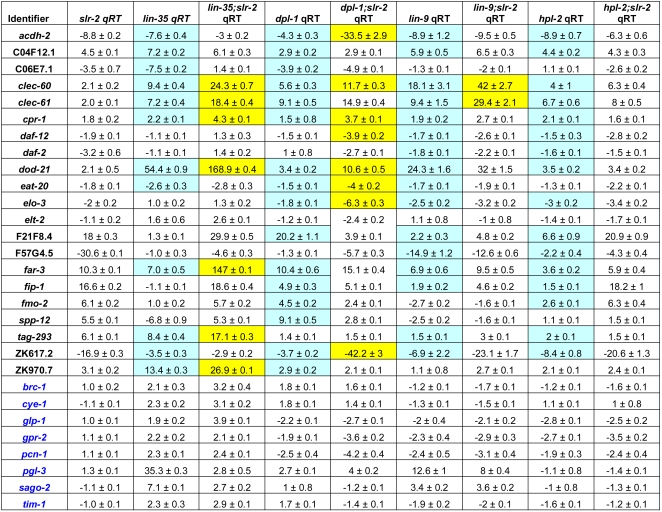
Transcriptional analysis in SynMuv mutants. qRT-PCR was used to quantitate the expression levels of 29 tester genes described in the text (also see Table S1). Data represent the mean of at least 4 samples along with their standard errors. Target genes displaying co-regulation between *slr-2* and individual SynMuv genes are highlighted in light blue. Genes showing synergistic misregulation in double mutants are highlighted in yellow. Note that a high frequency of synergistic misregulation is observed only in *lin-35;slr-2* and *dpl-1;slr-2* double mutants, which display strong synthetic genetic interactions (Table 1). Also note that both co-regulation and synergistic misregulation in *lin-35;slr-2* and *dpl-1;slr-2* mutants is limited to targets associated with intestinal and metabolic functions. For additional information, see text.

To gain mechanistic insight into the observed differences between the interactions of *slr-2* with *lin-35* and *dpl-1*, and the non-interacting SynMuv genes, we used qRT-PCR to assay expression levels of 29 genes identified previously by our transcriptome analysis. This included seven genes with intestine-specific expression, ten with known roles in energy production and metabolic regulation, and eight associated with cell cycle, germline, and RNAi functions (Table S2). We first examined expression levels in staged L1 single mutants to determine the frequency of co-regulation between *slr-2* and the SynMuv genes. We define co-regulation as the occurrence of a simultaneous increase or decrease in target gene expression levels by ≥1.5 fold relative to wild type. Interestingly, similar levels of co-regulation with *slr-2* were observed between the strong interacting (*lin-35* and *dpl-1*) and weak or non-interacting (*lin-9* and *hpl-2*) SynMuv genes tested; *lin-35*, *dpl-1*, *lin-9*, and *hpl-2* showed co-regulation of 12, 17, 16, and 17 genes, respectively (Figure 8). This result suggests that target co-regulation per se is not sufficient to cause a synthetic genetic interaction. Interestingly, co-regulation was only observed among the intestine-specific and metabolic regulator gene classes.

To better understand the basis of the observed genetic interactions, we performed qRT-PCR analysis in staged L1 double mutants (also see Materials and Methods). Specifically, we looked for synergistic misregulation effects on co-regulated genes identified by our analysis of single mutants. Genes were scored positive for synergistic effects if the fold change observed in double mutants was ≥1.95-fold higher than the greatest fold-change observed in either single mutant. Thus, for single mutants displaying fold changes of 3.0 and 6.0, a fold change of 11.7 or greater would be defined as synergistic (greater than additive). Notably, we observed synergistic effects on 7/12 and 8/17 *lin-35*–*slr-2* and *dpl-1*–*slr-2* co-regulated targets, respectively (Figure 8). In contrast, only 2/16 *lin-9*–*slr-2* and 0/17 *hpl-2*–*slr-2* co-regulated targets displayed synergistic misregulation (Figure 8). To see if the above differences were statistically significant, we applied a chi square test and found no significant difference between *lin-35*; *slr-2* and *dpl-1*; *slr-2* double mutants. Comparison of *lin-9*; *slr-2* and *hpl-2*; *slr-2* with *lin-35 slr-2* double mutants, however, showed that the observed differences were statistically significant (p = 0.01 and p<0.001, respectively). These results suggest that the strong synthetic phenotypes observed in *lin-35*; *slr-2* and *dpl-1*; *slr-2* double mutants may be due to the synergistic misregulation of common targets.

## Discussion

We have demonstrated a novel role for the pocket protein ortholog *lin-35/Rb* in nutrient utilization by the intestine. To our knowledge, these findings constitute the first direct demonstration of a role for pRb family members in the basic maintenance of organ functions. Furthermore, this function is carried out redundantly with SLR-2, a putative transcription factor and previously uncharacterized member of the *C. elegans* Zn-finger protein family. Multiple lines of evidence support the model that LIN-35 and SLR-2 act within intestinal cells to control the expression of genes required for the proper functioning of the digestive tract. (1) The early growth and developmental arrest of *lin-35*; *slr-2* mutants, coupled with an absence of morphological defects, is consistent with an inability to utilize nutrients (Figure 1, Figure S10). (2) Transcriptome profiling of *lin-35* and *slr-2* single mutants revealed extensive involvement in the regulation of intestine-associated genes (Figure 3; Figure S6). (3) Mosaic analysis demonstrated a requirement for both LIN-35 and SLR-2 within intestinal cells (Figure 4). (4) Based on UV-induced intestinal autofluorescence (Figure 6) and the DAF-16::GFP reporter (Figure 5), *lin-35*; *slr-2* larvae experience nutrient deprivation when grown on a standard bacterial food source. (5) The larval arrest of *lin-35*; *slr-2* mutants can be partially suppressed by growth on a processed synthetic medium. (6) Both *lin-35* and *slr-2* are expressed in cells of the intestine during early larval development and the expression of *slr-2* in the intestine is highly specific at this stage (Figure 7).

Currently, our data do not distinguish between specific classes of intestinal defects such as those affecting nutrient uptake, processing, or the dissemination of nutrients to other tissues. However, the sizeable number and diverse array of intestinal genes affected in both *lin-35* and *slr-2* mutants could indicate that multiple, and potentially additive, defects may be involved. We note that foregut-based defects are effectively ruled out as a primary cause of starvation in *lin-35*; *slr-2* mutants based on data from our mosaic analysis as well as additional results indicating that double mutants can both internalize and mechanically disrupt OP50 bacteria (Figures 11–12).

A central question posed by our analysis concerns the mechanistic basis of the observed synthetic genetic interaction between *lin-35* and *slr-2*. This question is most directly addressed by our studies of synthetic genetic interactions between *slr-2* and nine SynMuv genes (including *lin-35*), and by our subsequent analysis of transcriptional effects in a subset of both single and double mutants. Somewhat surprisingly, among the 29 genes assayed in single mutants, we found equivalent levels of transcriptional co-regulation between *slr-2* and the non-interacting SynMuv genes (*hpl-2* and *lin-9*) and between *slr-2* and the strong interactors (*lin-35* and *dpl-1*). This result suggests that regulon overlap alone is not sufficient for the induction of a synthetic genetic interaction (Table 1 and Figure 8). In contrast, only *lin-35*; *slr-2* and *dpl-1*; *slr-2* double mutants showed a high frequency of synergistic misregulation of common targets (Figure 8). This finding suggests that it is the synergistic misregulation of common targets that may specifically predispose animals to displaying strong synthetic phenotypes. It is also worth noting that enhanced effects on targets (equivalent to changes ≥1.95-fold) in double mutants were generally only observed in cases where both single mutants showed evidence of co-misregulation (Figure 8). Although these trends are compelling, our data does not address whether or not the synthetic phenotype occurs as a result of the synergistic misregulation of one or multiple genes. Certainly, the finding that a potentially sizeable number of genes are synergisitically misregulated in both *lin-35*; *slr-2* and *dpl-1*; *slr-2* mutants is consistent with a mechanism involving combined effects on many targets. Nevertheless, in the case of the SynMuv phenotype, it has been shown that it is the ultimate misregulation of a single gene, *lin-3*, that likely accounts for the excess in vulval cell induction [54].

It is also striking that of the seven Slr mutants identified by our laboratory, four (*spr-1*, *xnp-1*, *psa-1*, and *slr-2*) encode transcriptional regulators [27],[30],[31]. Furthermore, results from other laboratories reveal a strong overrepresentation of transcription factors among genes that display genetic redundancy with *lin-35/Rb*, including many of the discovered SynMuv genes [22],[25],[33],[34],[55]. Thus, a common theme among *lin-35*-synthetic interactors appears to be transcriptional regulation. These findings are further consistent with large-scale screens in *S. cerevisiae* indicating that genetic redundancy is more frequently observed between genes with similar predicted molecular functions [56], and suggests that similar trends will be observed in *C. elegans* and higher organisms.

Our discovery of a role for LIN-35 in promoting nutrient utilization by the intestine also complements several recent reports describing roles for pRb family members in intestinal cell proliferation, morphogenesis, and differentiation. Recently, Haigis and coworkers reported that that simultaneous loss of pRb and either p107 or p130 leads to both structural aberrations and incomplete cellular differentiation in intestinal villi [57]. There are conflicting reports, however, as to whether single mutations in pocket protein members are sufficient to disrupt intestinal development [57]–[59]. Notably, one study using a conditional Rb knockout approach observed dramatic hyperproliferation of intestinal epithelium, leading to villi filling up the luminal space [59]. Consistent with this, we and others have observed pronounced hyperproliferation of intestinal nuclei in double mutants of *lin-35* and either *fzr-1* [20] or *cki-1* [17]. In contrast to the above studies, our current findings uniquely implicate LIN-35 in nutrient acquisition or utilization by the intestine (organ function), and show that this defect is not due to overt cell cycle abnormalities. This latter finding is also consistent with an absence of cell cycle genes from the *slr-2* regulon (Figure 3; Figure S6). Interestingly, the human ortholog of another *lin-35*-synthetic gene identified by our screen, *xnp-1*/ATR-X, was recently implicated in intestinal functions, as mutations in ATR-X led to a wide range of gastrointestinal abnormalities [60].

Our study also further links Zn-finger proteins to pRb-related functions, as a sizeable number of C2H2-type proteins have been shown to physically or genetically interact with pocket protein family members [27], [34], [55], [61]–[66]. In *C. elegans*, *lin-35*-interacting Zn-finger transcription factors include *lsy-2* and *zfp-2*, which function coordinately with *lin-35* to promote fertility [55]; *mcd-1*, which acts with *lin-35* to promote apoptosis [34]; and *spr-4,* which redundantly regulates vulval morphogenesis with *lin-35* [27]. In addition, the THAP-domain protein GON-14 functions redundantly with LIN-35 to promote larval growth [33], and a mammalian THAP-domain protein, THAP1, acts in parallel to pRb/E2F to control the expression of E2F target genes required for G1–S-phase progression [66]. Thus, interactions between LIN-35 and Zn-finger proteins (frequently C2H2-type proteins) in various organisms have important and versatile roles in development.

## Materials and Methods

### Strains and Maintenance

*C. elegans* strains were maintained according to established procedures [67], and all experiments were carried out at 20°C. Strains used in these studies include the following: N2, wild type; MT10430, [*lin-35*(*n745*) further backcrossed by our laboratory 5×] [25]; MH1461, [*lin-35*(*n745*); *kuEx119*]; MH1620, [*lin-35*; *slr-2(ku297)*; *kuEx119*]; MT1799, [*lin-36*(*n766*); *unc-32*(*e189*)]; MT5470, [*lin-37*(*n758*)]; MT1806, [*lin-15A*(*n767*)]; MT2495, [*lin-15B*(*n744*)]; MT8840, [*dpy-5*(*e61*); *lin-53*(*n833*)]; MT8879, [*dpl-1*(*n2994*)]; PFR40, [*hpl-2*(*tm1489*)]; TJ356, [*daf-*16::GFP; *rol-6*]; TY903, [*yDf7/unc-76*(*e911*)]; WY53, [*lin-35*(*n745*); *unc-76*(*e911*); *rol-9*(*sc148*)]; WY286, [*slr-2*(*ku297*); *fdEx25* (CBG05648+*sur-5*::GFP)]; WY447, [*lin-35*(*n745*); *slr-2*(*ku297*); *fdEx25*]; WY471, [*lin-35*; *daf-*16::GFP; *rol-6*]; WY472, [*daf-*16::GFP; *rol-6*; *slr-2*]; WY473, [*lin-35*; *daf-*16::GFP; *rol-6*; *slr-2*; *kuEx119*].

### *slr-2(ku297)* Genetic Mapping

Genetic mapping of the *slr-2(ku297)* locus was performed using established procedures [for details see [68]]. Briefly, *slr-2* was mapped between *unc-76* and *rol-9* on LGV. SNP mapping (using the online SNP database at http://genome.wustl.edu/genome/celegans/celegans_snp.cgi) was then used to place the *slr-2* locus in an 82.3-kb region defined by SNP:Y59A8B:89830 and SNP:Y59A8B:172162, which contains nine predicted genes.

### *C. briggsae slr-2* Transgenic Rescue

The following primers were used to amplify the *C. briggsae slr-2* ortholog, CBG05648: 5′-GTGGCATTGTAGGACGATACCC-3′ and 5′-GGAATTCGGAGGGAATTTGAAC-3′. The resulting PCR product, together with the *sur-5*::RFP marker, was injected into *lin-35*; *slr-2*; *kuEx119* mutants to generate lines carrying an RFP-marked extrachromosomal array. Rescue was inferred by the ability of the RFP-encoding array to confer viability to worms lacking the GFP-marked *lin-35* rescuing array (*kuEx119*). Seven of seven lines isolated from independently injected P0s yielded strains that could be propagated in the absence of *kuEx119*.

### *slr-2* Gene Structure

3′ RACE was performed using domains of Y59A8B.13 that are conserved between *C. elegans*, *C. briggsae*, and *C. remanei*. 5′ RACE was performed using a primer complementary to the SL1 trans-splice leader sequence and a primer specific to Y59A8B.13.

### Microarray, qRT-PCR, and Statistical Analysis

RNA was extracted from staged L1 larvae, purified, and used for microarray analysis as previously described [21]. Differentially expressed genes were identified by comparison with identically staged N2 worms using RMA software, as previously described [21]. RNA was extracted from staged L1 larvae, purified, and used for qRT-PCR as previously described [21]. Intestine-specific/enriched genes referenced in our studies were obtained from a previously published SAGE analysis and displayed a minimum intestine:whole worm tag ratio of >2.6 as previously determined [41]. Our P-values were calculated using either t-tests or chi-square tests, where appropriate, using the statistic language R. Pearson correlation coefficients between *lin-35* and *slr-2* coregulated genes as well as standard errors of mean (or deviation) for all other experiments was calculated using Microsoft Excel. A staged (L1) population of *lin-35*; *slr-2* double mutants (lacking the *kuEx119* array) was obtained using a COPAS worm sorter from Union Biometrica.

### *slr-2::GFP*

A transcriptional reporter was generated by amplifying an ∼900-bp region upstream of the *slr-2* start codon using the following primers: 5′-CCCATTATCGGCCATTTTTGCTG-3′ and 5′-GGTGCAGGTCGACACTTTTCGACATTTCCGGTGGTCTG-3′. Based on the location of the confirmed gene, Y59A8B.12, which is located <1 kb upstream of the translational start site for *slr-2*, this sequence is likely to encompass the complete 5′ regulatory region of *slr-2*. Following digestion with *Xho*I and *Sal*I, the resulting PCR product was inserted into pPD95.69 (gift of A. Fire), and the obtained plasmid was injected in N2 animals to establish multiple independent extrachromosomal arrays, all of which showed similar patterns of GFP expression. One extrachromosomal array line was then chosen for integration using standard irradiation methods [69].

### *daf-18(RNAi)*

RNAi was carried out using standard procedures [70]. MH1620 L4 hermaphrodites were placed on *daf-18(RNAi)* feeding plates and F1 progeny were assayed 4–5 days later for gonad size.

## Supporting Information

Figure S1 *slr-2* cDNA and predicted translated peptide. For additional details, see Materials and Methods and text.
(2.49 MB TIF)

Figure S2 SLR-2-responsive genes. A complete list of the 1736 genes that showed differential expression in *a slr-2* mutant strain. The column labeled “Probesets” refers to Affymetrix probe set numbers, the column referred to as “mountain” refers to gene mountains, described in Kim, et al, 2001.
(0.27 MB XLS)

Figure S3 Correlation between microarray and qRT-PCR for *slr-2*-responcive genes. qRT-PCR and microarray data for *slr-2*-responsive genes show similar changes in differential expression. Numbers for qRT-PCR represent the mean of at least four samples along with their standard errors. Genes shown in blue include cell cycle, germline, and RNAi-associated genes.
(0.34 MB TIF)

Figure S4 SLR-2-responsive genes in an adult SAGE gut library. A complete list of the 261 genes that were differentially expressed in *slr-2* mutants as well as present in a SAGE library prepared from the intestine of adult worms (Figure 3; McGhee, et al, 2007). The column labeled “Probesets” refers to Affymetrix probe set numbers, the column “Fold Change” represents the level of differential expression in a *slr-2* mutant background compared to wild type. The column labeled “mountain” refers to gene mountains, described in Kim, et al, 2001.
(0.05 MB XLS)

Figure S5 SLR-2-responsive genes differentially expressed in *lin-35* mutant worms. A complete list of 261 genes that were differentially expressed in *slr-2* mutants as well as in *lin-35* mutants (Figure 3). The column labeled “Probesets” refers to Affymetrix probe set numbers. The column “Fold Change” represents the level of differential expression in a *slr-2* mutant background. The column labeled “mountain” refers to gene mountains, described in Kim, et al, 2001.
(0.04 MB XLS)

Figure S6 Genes from categories I-IV exhibit strong enrichment in genes associated with intestine and metabolic functions. (A) Overlapping genes from categories I-IV display specific overrepresentation of intestine and metabolic mountains. (B, C) Available expression database results showing that a majority of category I-IV genes exhibit intestinal expression, including many that are intestinal specific. (D) Genes from categories I-IV are enriched for GATA sites relative to the SAGE gut dataset (also see Materials and Methods and Results).
(1.67 MB TIF)

Figure S7 *lin-35*; *slr-2* cell cycle analysis. (A) Graph showing average intestinal nuclei numbers in wild type (N2), *slr-2*, *lin-35*, *lin-35*; *slr-2*, and *fzr-1* mutants (n = 50 for each strain). Bars indicate standard deviations. Differences observed between *lin-35*; *slr-2* and *fzr-1* mutants with wild type were statistically significant (p<0.001). (B) Graph showing distribution of intestinal nuclei numbers in wild-type and mutant strains. Note that whereas only 46% of *lin-35*; *slr-2* double mutants contain greater numbers of intestinal nuclei than wild type, 100% of double mutants arrest. In contrast, whereas 72% of *fzr-1* mutants contain extra nuclei, only 2% undergo arrest. DAPI staining of intestinal nuclei in N2 (C) and *lin-35*; *slr-2* double mutants (D). DAPI staining was measured for 25 nuclei (from 8 worms for each genotype) using Openlab software. N2 worms exhibited average fluorescence values of 125% of background and *lin-35*; *slr-2* double mutants exhibited average fluorescence values 127% of background. Arrowheads illustrate intestinal nuclei. Scale bar: 10 µm in panels C and D.
(1.34 MB TIF)

Figure S8 Intestinal UV autofluorescence in wild-type, *lin-35*, *slr-2*, and *lin-35*; *slr-2* double mutant larvae. (A) Panels show representative images (under DAPI channel UV) of intestines from well-fed or starved wild-type, *lin-35*, *slr-2*, and *lin-35*; *slr-2* of synchronized larvae. (B) Quantitiation of autofluorescence patterns corresponding to Panel A. Fluorescence was assigned as either punctuate (punct) or diffuse (dif). Note that qualitative differences can be detected between *lin-35*; *slr-2* larvae and other tested strains as early as three hours, though maximal effects are observed by twelve hours. Scale bar: in A, 10 µm for all panels.
(8.36 MB TIF)

Figure S9 DAF-16::GFP expression in *lin-35*; *slr-2* mutants rescued for arrest by growth on axenic media. DIC (A) and corresponding DAF-16::GFP fluorescence (B) micrographs of a *lin-35*; *slr-2* double mutant rescued from arrest by growth on synthetic axenic media. Note that DAF-16::GFP shows a diffuse cytosolic (non-nuclear) pattern of localization in the intestine, similar to fed wild-type animals. Also see Figure 5. Scale bar: 10 µm for both panels.
(1.02 MB TIF)

Figure S10 DAF-18 mediates developmental arrest in *lin-35*; *slr-2* double mutants. DIC micrographs of *lin-35*; *slr-2* double mutants on OP50 (A, B) or *daf-18(RNAi)* feeding plates (C, D). Animals imaged were the progeny of fertile *lin-35(n745)*; *slr-2(ku297)*; *kuEx119* mutants in which the extrachromosomal array had been lost (also see Materials and Methods). Representative gonad sizes (A, C) as well as the largest gonads observed (B, D) for both OP50 and *daf-18(RNAi)*-treated animals. Gonads are outlined with a yellow dashed line. Scale bar: 10 µm for A-D. (E) Quantification of gonad size in starved WT (N2), *lin-35;slr-2* and *lin-35;slr-2; daf-18(RNAi)* animals. Error bars represent standard deviation. Note that *daf-18(RNAi)* leads to an ∼2-fold increase in the average size of gonads.
(4.44 MB TIF)

Figure S11 *lin-35*; *slr-2* larvae can internalize food analogs. (A-C) Following synchronization on plates without food, wild-type (N2), *lin-35*, *slr-2*, and *lin-35*; *slr-2* double mutants were cultured on plates in the presence (fed) or absence (st) of OP50 bacteria for the times shown (A-C). At the indicated time points, larvae were transferred to plates containing a visually detectable food analog (fluorescent beads, Polyscience, Inc., FluoresbriteTM Polychromatic red microspheres, CAT#19507) for 30 minutes, and bead internalization was then scored by fluorescence microscopy in 50–70 larvae for each time point. Note that at all time points observed, *lin-35*; *slr-2* double mutants did not vary significantly from single mutant controls, nor did fed and starved populations vary significantly from each other. DIC (D, F) and corresponding GFP (E, G) micrographs of wild-type (D, E) and *lin-35*; *slr-2* double mutants (F, G) scored as capable of internalizing beads at 12 hours. Scale bar: 10 µm for panels D-G.
(2.12 MB TIF)

Figure S12 *lin-35*; *slr-2* larvae can mechanically disrupt bacteria. Following synchronization on plates without food, wild-type (N2) and *lin-35*; *slr-2* larvae were cultured in the absence of food for an additional 3 to 36 hours before placement on plates containing a GFP-marked OP50 bacterial strain. Mechanical disruption is indicated by the presence of GFP-fluorescing bacteria in the foregut only (A). Even after extended periods of time (24–36 hours), the majority of *lin-35*; *slr-2* double mutants were capable of disrupting OP50. Furthermore, mechanical disruption in *lin-35*; *slr-2* larvae was indistinguishable from wild type at 12 hours, where other assays showed clear indications of starvation (also see main text). DIC (B, D) and corresponding GFP (C, E) micrographs of *lin-35*; *slr-2* double mutants that have ingested GFP-marked OP50 bacteria (OP50-GFP strain). Panels B and C depict representative images obtained for the majority of assayed larvae, where GFP fluorescence can be detected only in regions of the alimentary canal that are anterior to the posterior pharyngeal bulb (grinder), where the mechanical disruption of bacteria normally occurs. In a minority of worms at the 24 and 36 hour time points (D and E), some fluorescent bacteria observed in the intestine (arrow). Pharyngeal regions are delineated by yellow braces. Scale bar: 10 µm in panels B-E.
(2.16 MB TIF)

Figure S13 Expression of *slr-2*::GFP. DIC (A, C, E) and corresponding GFP (B, D, F) micrographs of an L4 larvae (A, B), a pre-morphogenetic embryo (∼300 cell stage; C, D), and late-stage embryo (E, F). Although brightest in the intestinal posterior, reporter expression was observed throughout the intestine in L4 stage larvae. In addition, L4 larvae exhibited expression in marginal cells and the m3VR, mC, I5, m5, m6, and m7 cells of the pharynx, as well as a small subset of head neurons and the excretory duct cell (A, B). Ubiquitous expression was observed in the early embryo (C, D), which becomes largely restricted to the intestine by the pretzel-stage, where expression is brightest in the posterior gut region (E, F). In panels A and B, the posterior region displaying the greatest intense intestinal fluorescence is delineated by a solid white brace and the adjacent dimmer region is delineated by a white, dashed brace. The foregut is delineated by a white dotted dashed brace. In panels E and F, the posterior intestinal region is circled. Scale bar: A, B 100 µm, C-F, 10 µm.
(7.21 MB TIF)

Table S1 Insulin and TOR signaling networks are down regulated in *slr*-2 mutants.
(0.05 MB DOC)

Table S2 Description of tester genes used for qRT-PCR analysis.
(0.05 MB DOC)
