# Supplementary figures and images for: Coordinated Regulation of Intestinal Functions in C. elegans by LIN-35/Rb and SLR-2
Source: PLoS Genet. 2008 Apr 25;4(4):e1000059. doi: 10.1371/journal.pgen.1000059 (PMC2312330; doi:10.1371/journal.pgen.1000059)

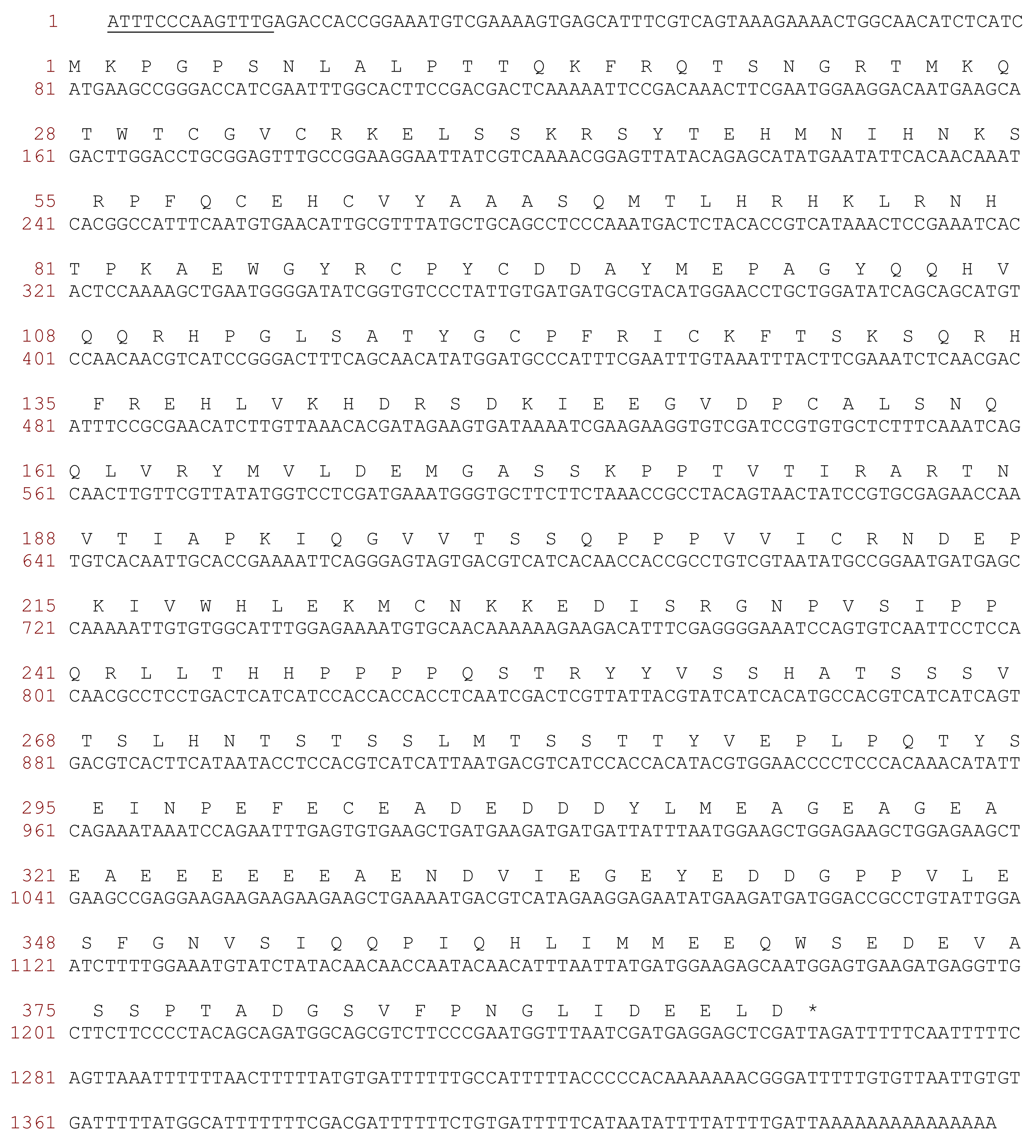

Supplement: Figure S1 — slr-2 cDNA and predicted translated peptide. For additional details, see Materials and Methods and text. (2.49 MB TIF) [file pgen.1000059.s001.tif]

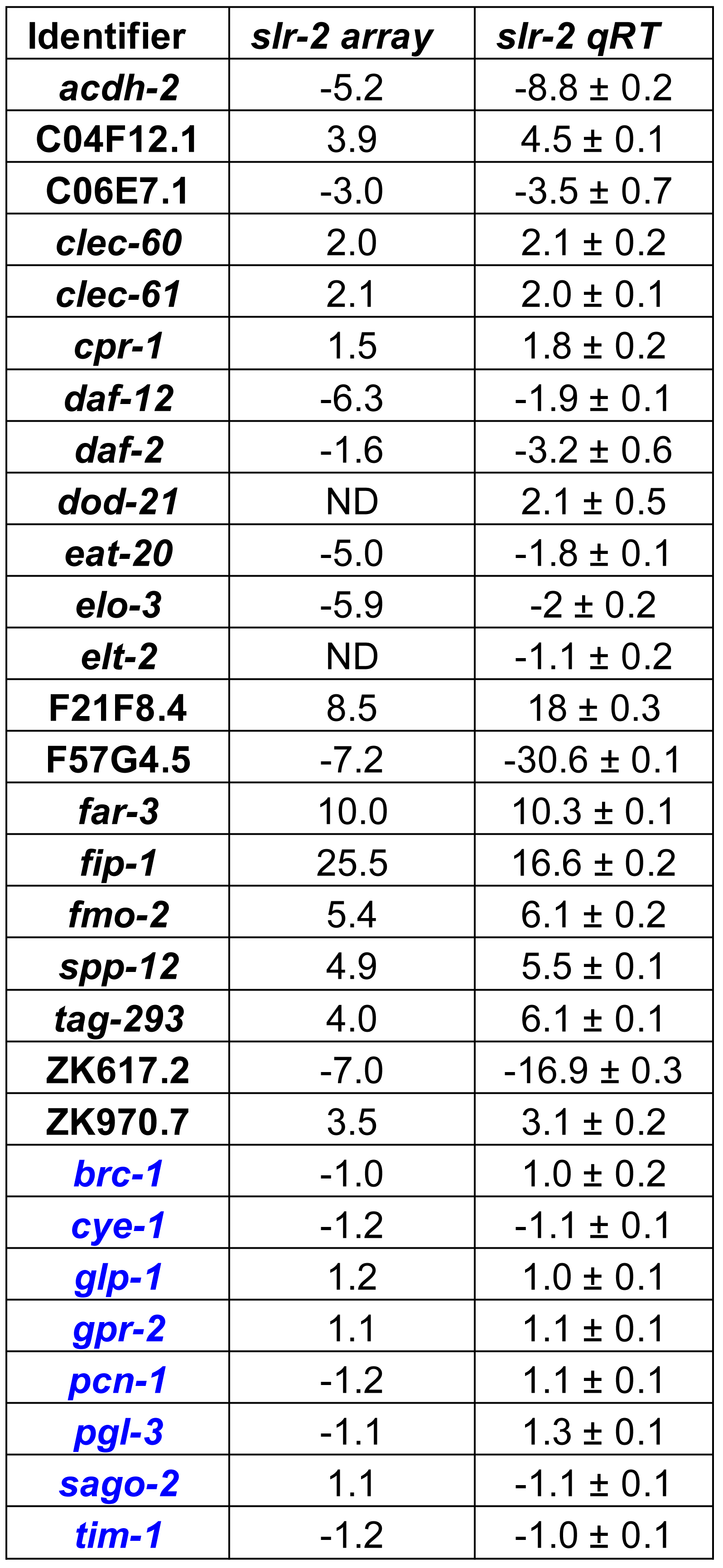

Supplement: Figure S3 — Correlation between microarray and qRT-PCR for slr-2-responcive genes. qRT-PCR and microarray data for slr-2-responsive genes show similar changes in differential expression. Numbers for qRT-PCR represent the mean of at least four samples along with their standard errors. Genes shown in blue include cell cycle, germline, and RNAi-associated genes. (0.34 MB TIF) [file pgen.1000059.s003.tif]

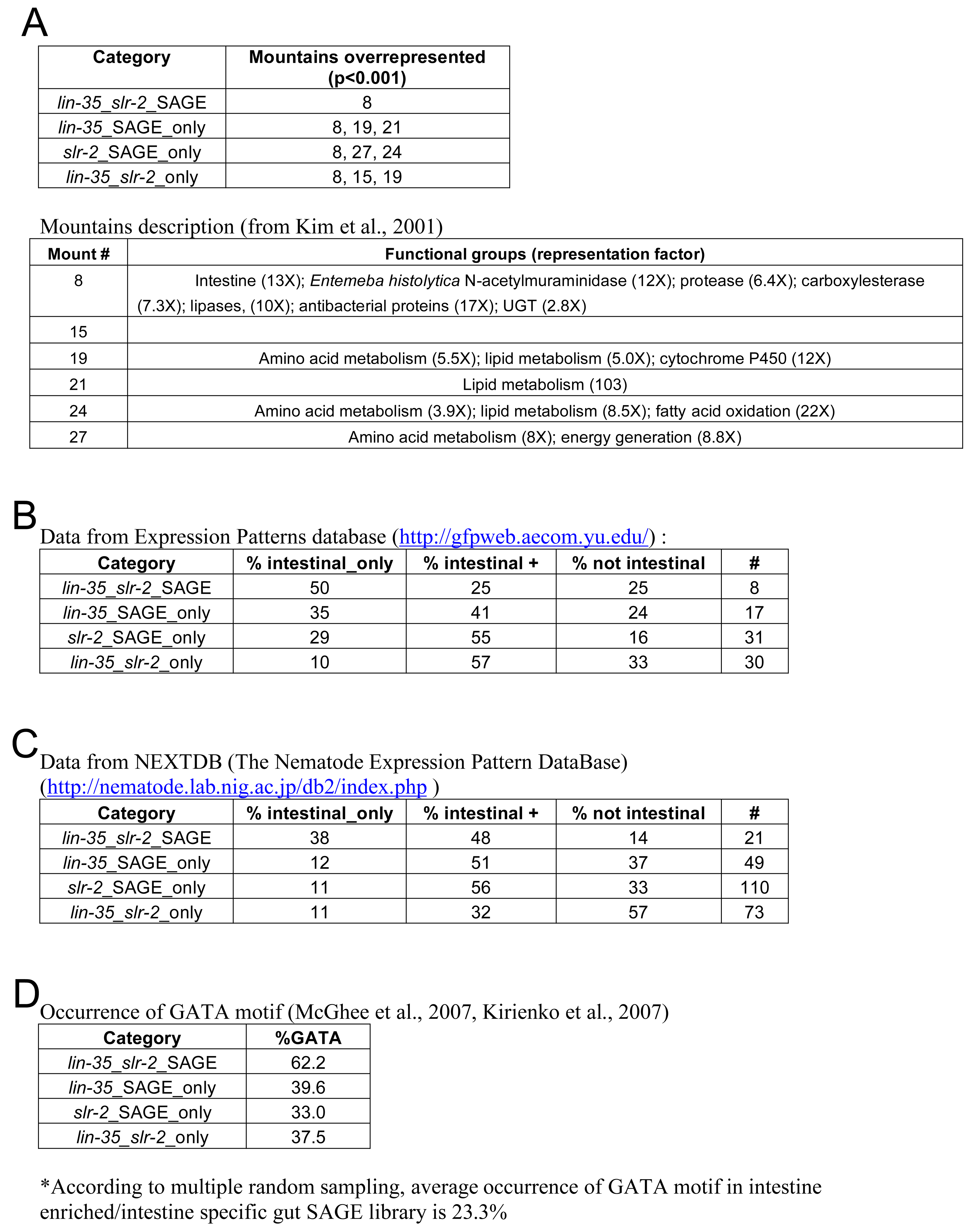

Supplement: Figure S6 — Genes from categories I-IV exhibit strong enrichment in genes associated with intestine and metabolic functions. (A) Overlapping genes from categories I-IV display specific overrepresentation of intestine and metabolic mountains. (B, C) Available expression database results showing that a majority of category I-IV genes exhibit intestinal expression, including many that are intestinal specific. (D) Genes from categories I-IV are enriched for GATA sites relative to the SAGE gut dataset (also see Materials and Methods and Results). (1.67 MB TIF) [file pgen.1000059.s006.tif]

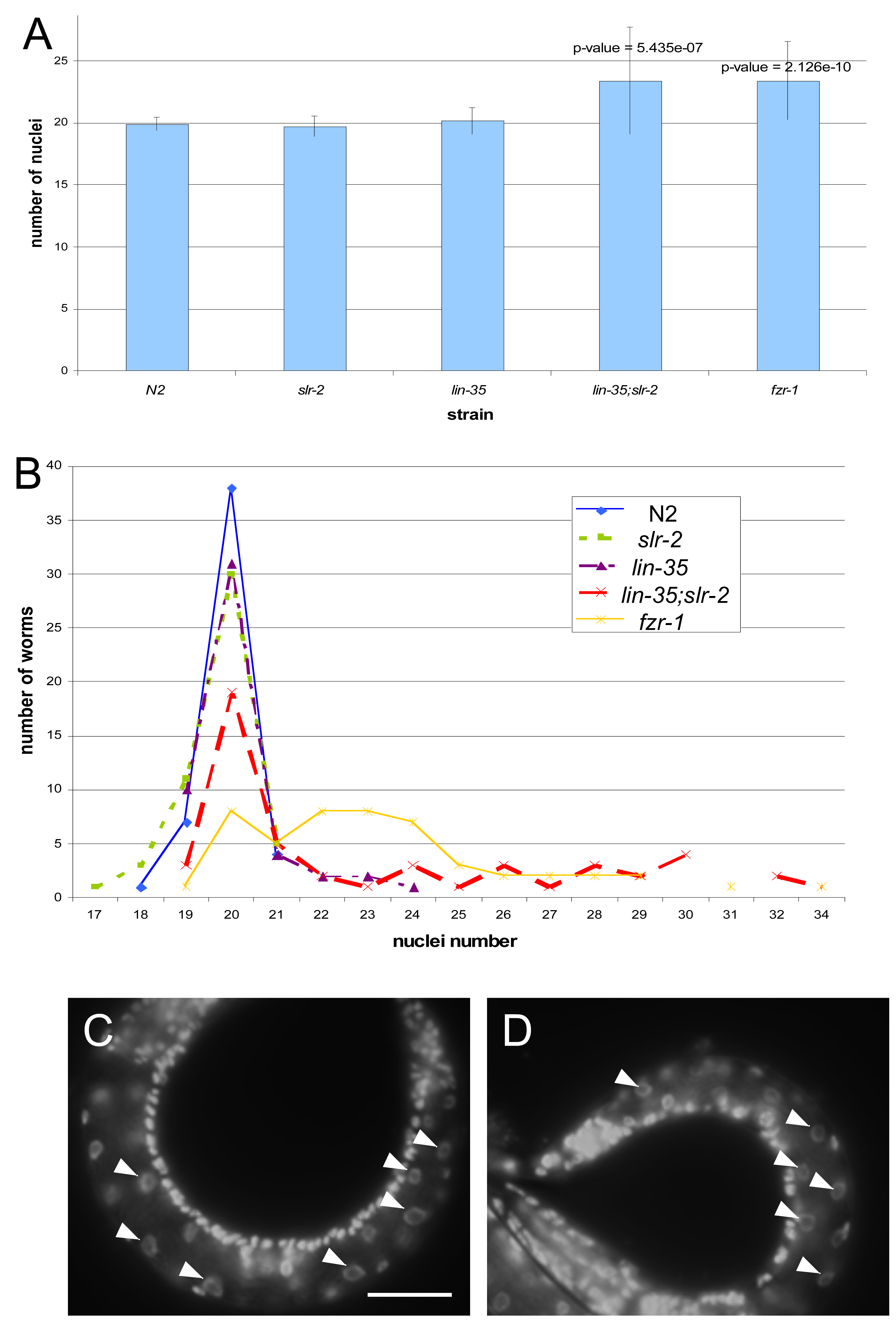

Supplement: Figure S7 — lin-35; slr-2 cell cycle analysis. (A) Graph showing average intestinal nuclei numbers in wild type (N2), slr-2, lin-35, lin-35; slr-2, and fzr-1 mutants (n = 50 for each strain). Bars indicate standard deviations. Differences observed between lin-35; slr-2 and fzr-1 mutants with wild type were statistically significant (p<0.001). (B) Graph showing distribution of intestinal nuclei numbers in wild-type and mutant strains. Note that whereas only 46% of lin-35; slr-2 double mutants contain greater numbers of intestinal nuclei than wild type, 100% of double mutants arrest. In contrast, whereas 72% of fzr-1 mutants contain extra nuclei, only 2% undergo arrest. DAPI staining of intestinal nuclei in N2 (C) and lin-35; slr-2 double mutants (D). DAPI staining was measured for 25 nuclei (from 8 worms for each genotype) using Openlab software. N2 worms exhibited average fluorescence values of 125% of background and lin-35; slr-2 double mutants exhibited average fluorescence values 127% of background. Arrowheads illustrate intestinal nuclei. Scale bar: 10 µm in panels C and D. (1.34 MB TIF) [file pgen.1000059.s007.tif]

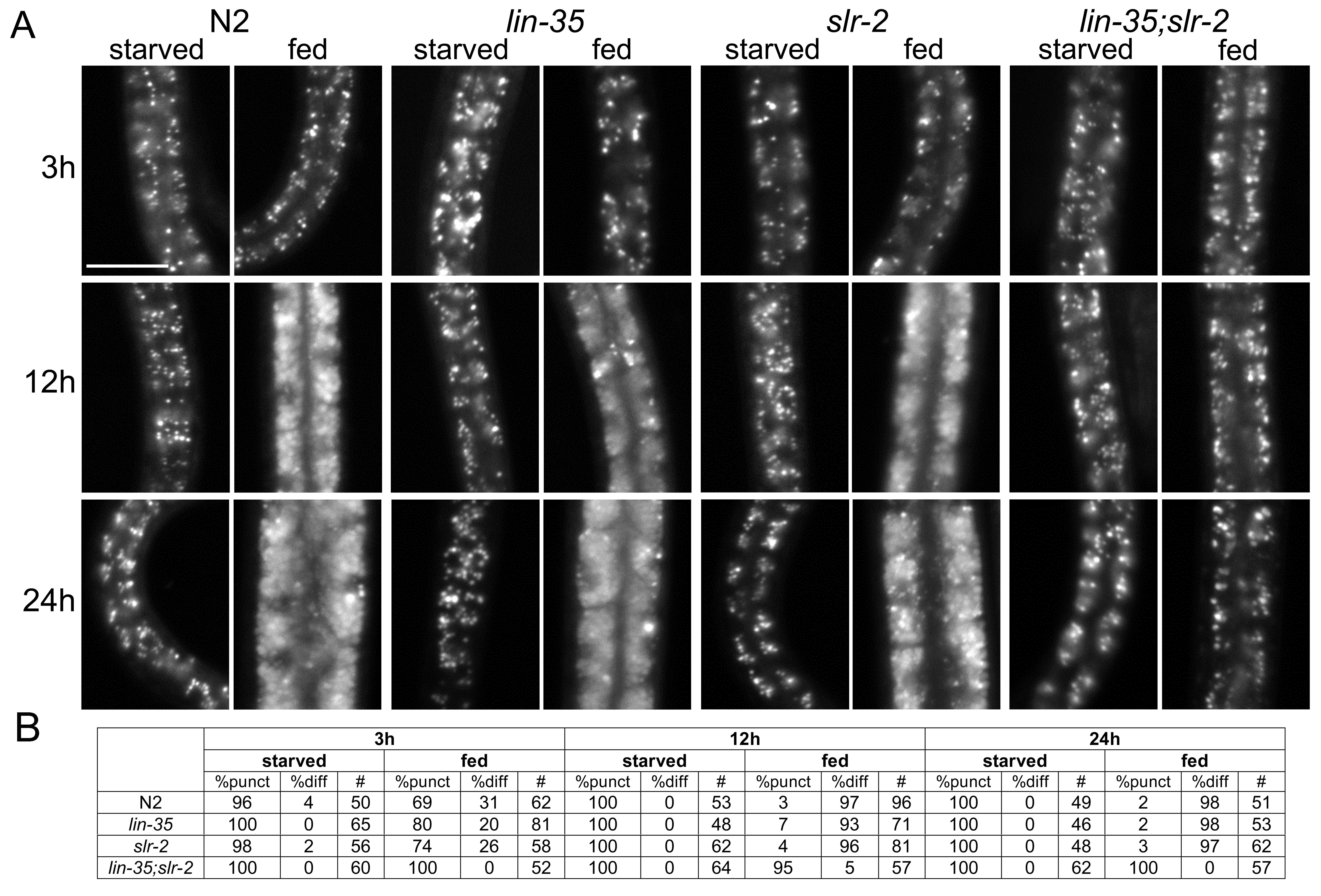

Supplement: Figure S8 — Intestinal UV autofluorescence in wild-type, lin-35, slr-2, and lin-35; slr-2 double mutant larvae. (A) Panels show representative images (under DAPI channel UV) of intestines from well-fed or starved wild-type, lin-35, slr-2, and lin-35; slr-2 of synchronized larvae. (B) Quantitiation of autofluorescence patterns corresponding to Panel A. Fluorescence was assigned as either punctuate (punct) or diffuse (dif). Note that qualitative differences can be detected between lin-35; slr-2 larvae and other tested strains as early as three hours, though maximal effects are observed by twelve hours. Scale bar: in A, 10 µm for all panels. (8.36 MB TIF) [file pgen.1000059.s008.tif]

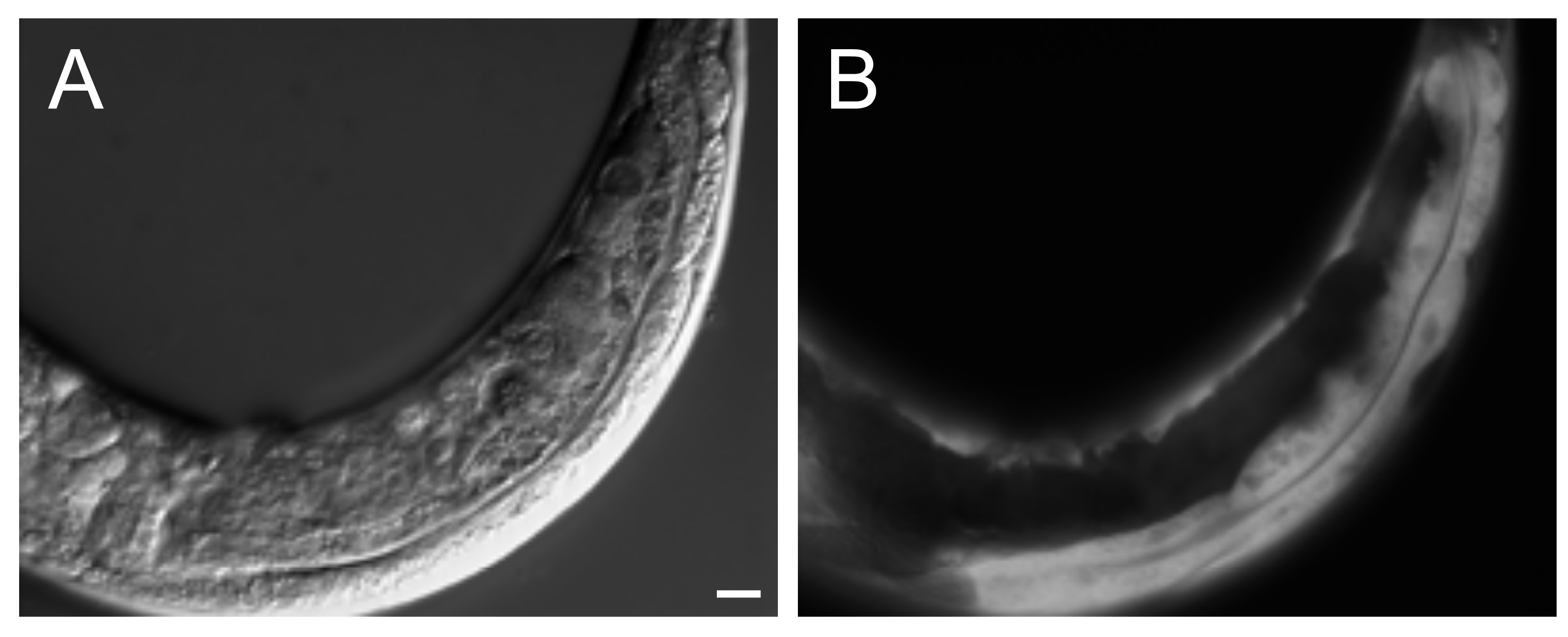

Supplement: Figure S9 — DAF-16::GFP expression in lin-35; slr-2 mutants rescued for arrest by growth on axenic media. DIC (A) and corresponding DAF-16::GFP fluorescence (B) micrographs of a lin-35; slr-2 double mutant rescued from arrest by growth on synthetic axenic media. Note that DAF-16::GFP shows a diffuse cytosolic (non-nuclear) pattern of localization in the intestine, similar to fed wild-type animals. Also see Figure 5. Scale bar: 10 µm for both panels. (1.02 MB TIF) [file pgen.1000059.s009.tif]

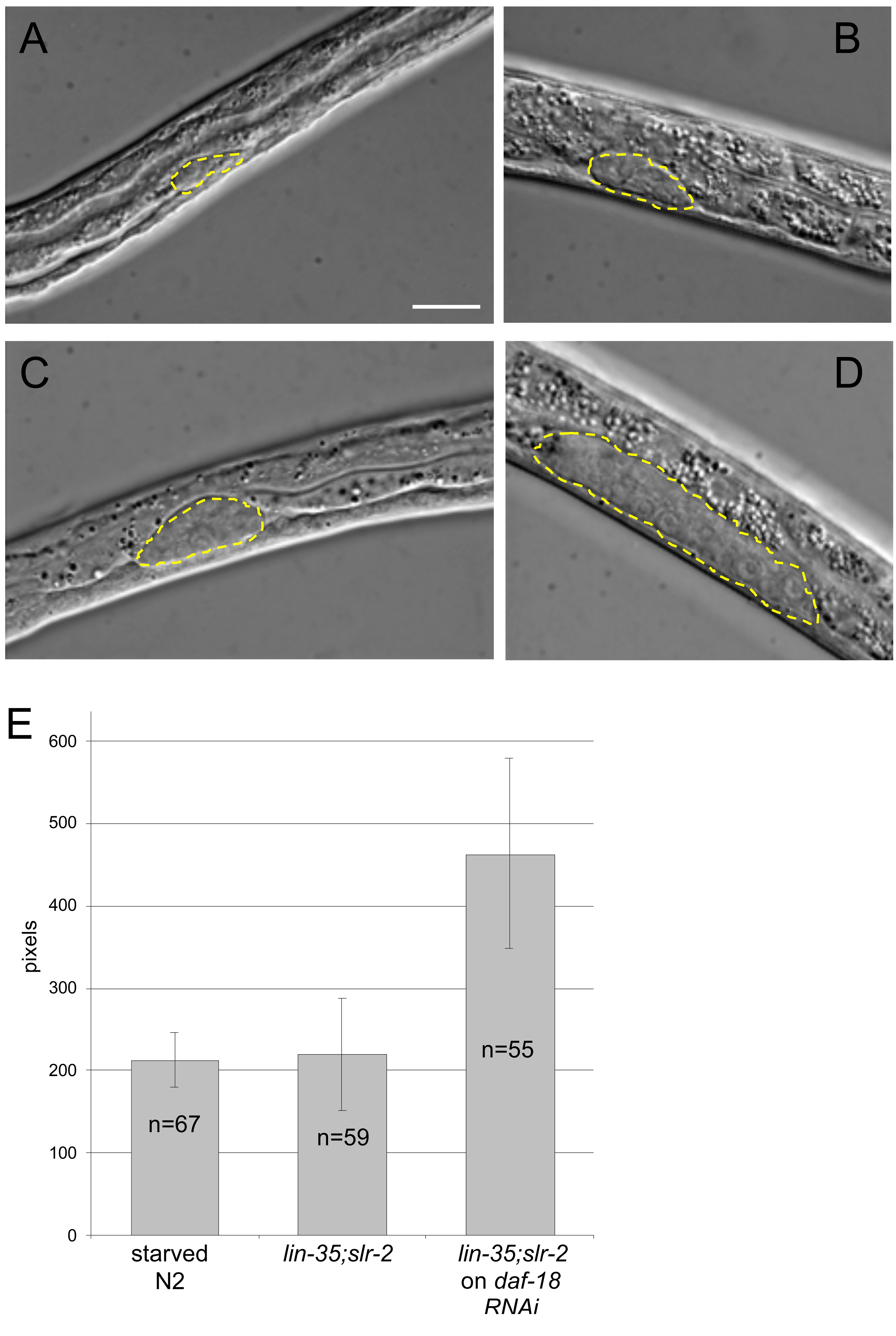

Supplement: Figure S10 — DAF-18 mediates developmental arrest in lin-35; slr-2 double mutants. DIC micrographs of lin-35; slr-2 double mutants on OP50 (A, B) or daf-18(RNAi) feeding plates (C, D). Animals imaged were the progeny of fertile lin-35(n745); slr-2(ku297); kuEx119 mutants in which the extrachromosomal array had been lost (also see Materials and Methods). Representative gonad sizes (A, C) as well as the largest gonads observed (B, D) for both OP50 and daf-18(RNAi)-treated animals. Gonads are outlined with a yellow dashed line. Scale bar: 10 µm for A-D. (E) Quantification of gonad size in starved WT (N2), lin-35;slr-2 and lin-35;slr-2; daf-18(RNAi) animals. Error bars represent standard deviation. Note that daf-18(RNAi) leads to an ∼2-fold increase in the average size of gonads. (4.44 MB TIF) [file pgen.1000059.s010.tif]

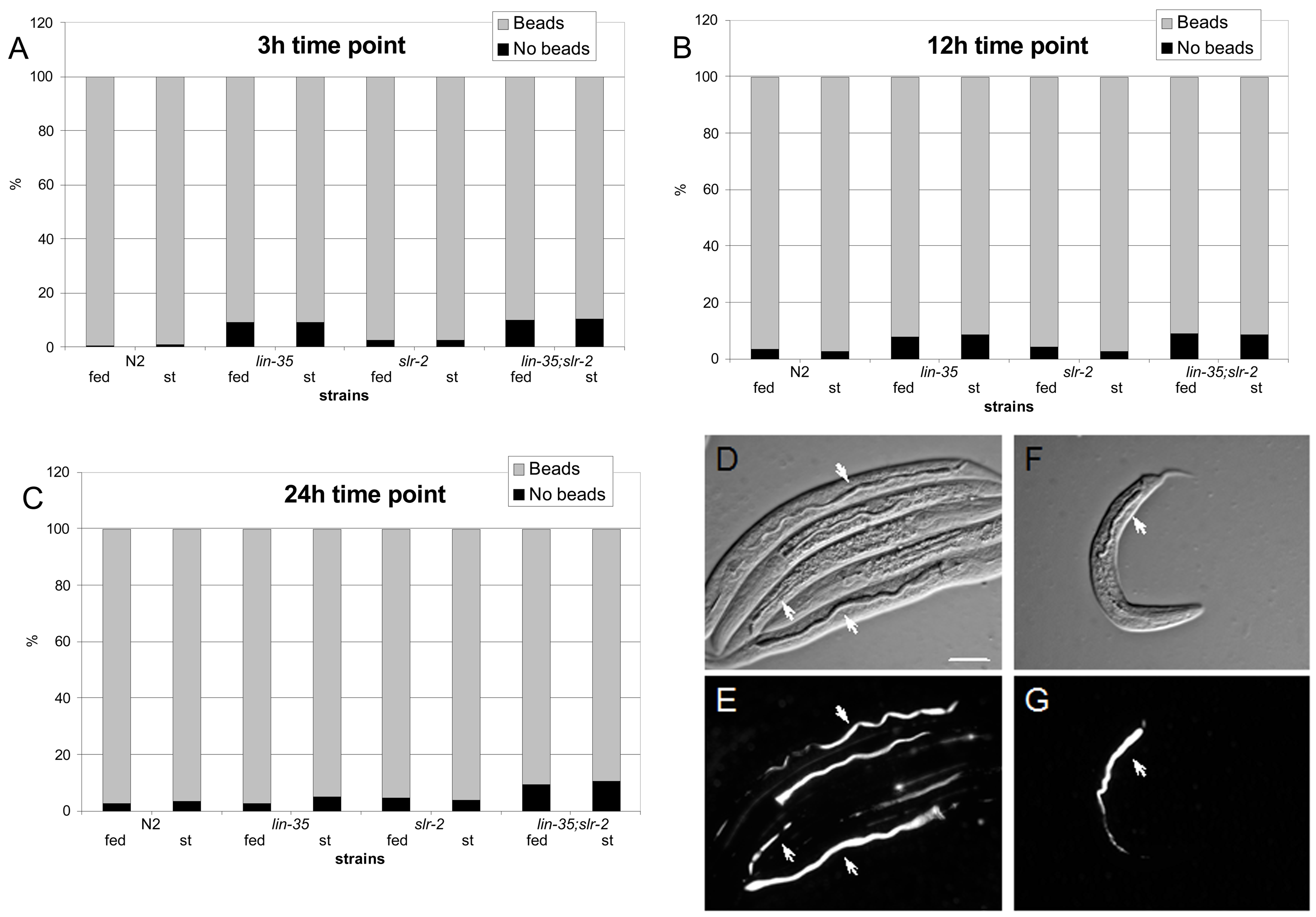

Supplement: Figure S11 — lin-35; slr-2 larvae can internalize food analogs. (A-C) Following synchronization on plates without food, wild-type (N2), lin-35, slr-2, and lin-35; slr-2 double mutants were cultured on plates in the presence (fed) or absence (st) of OP50 bacteria for the times shown (A-C). At the indicated time points, larvae were transferred to plates containing a visually detectable food analog (fluorescent beads, Polyscience, Inc., FluoresbriteTM Polychromatic red microspheres, CAT#19507) for 30 minutes, and bead internalization was then scored by fluorescence microscopy in 50–70 larvae for each time point. Note that at all time points observed, lin-35; slr-2 double mutants did not vary significantly from single mutant controls, nor did fed and starved populations vary significantly from each other. DIC (D, F) and corresponding GFP (E, G) micrographs of wild-type (D, E) and lin-35; slr-2 double mutants (F, G) scored as capable of internalizing beads at 12 hours. Scale bar: 10 µm for panels D-G. (2.12 MB TIF) [file pgen.1000059.s011.tif]

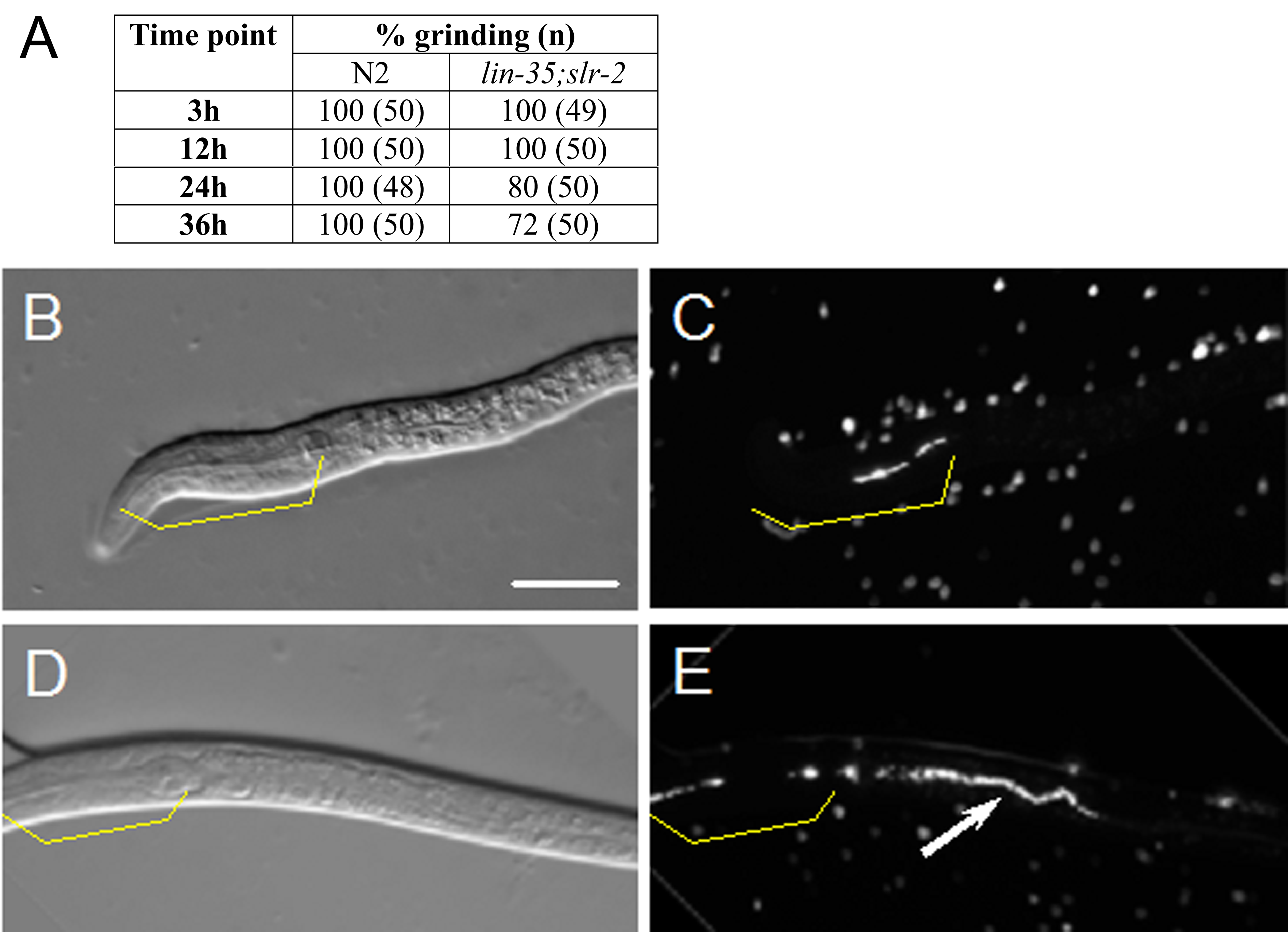

Supplement: Figure S12 — lin-35; slr-2 larvae can mechanically disrupt bacteria. Following synchronization on plates without food, wild-type (N2) and lin-35; slr-2 larvae were cultured in the absence of food for an additional 3 to 36 hours before placement on plates containing a GFP-marked OP50 bacterial strain. Mechanical disruption is indicated by the presence of GFP-fluorescing bacteria in the foregut only (A). Even after extended periods of time (24–36 hours), the majority of lin-35; slr-2 double mutants were capable of disrupting OP50. Furthermore, mechanical disruption in lin-35; slr-2 larvae was indistinguishable from wild type at 12 hours, where other assays showed clear indications of starvation (also see main text). DIC (B, D) and corresponding GFP (C, E) micrographs of lin-35; slr-2 double mutants that have ingested GFP-marked OP50 bacteria (OP50-GFP strain). Panels B and C depict representative images obtained for the majority of assayed larvae, where GFP fluorescence can be detected only in regions of the alimentary canal that are anterior to the posterior pharyngeal bulb (grinder), where the mechanical disruption of bacteria normally occurs. In a minority of worms at the 24 and 36 hour time points (D and E), some fluorescent bacteria observed in the intestine (arrow). Pharyngeal regions are delineated by yellow braces. Scale bar: 10 µm in panels B-E. (2.16 MB TIF) [file pgen.1000059.s012.tif]

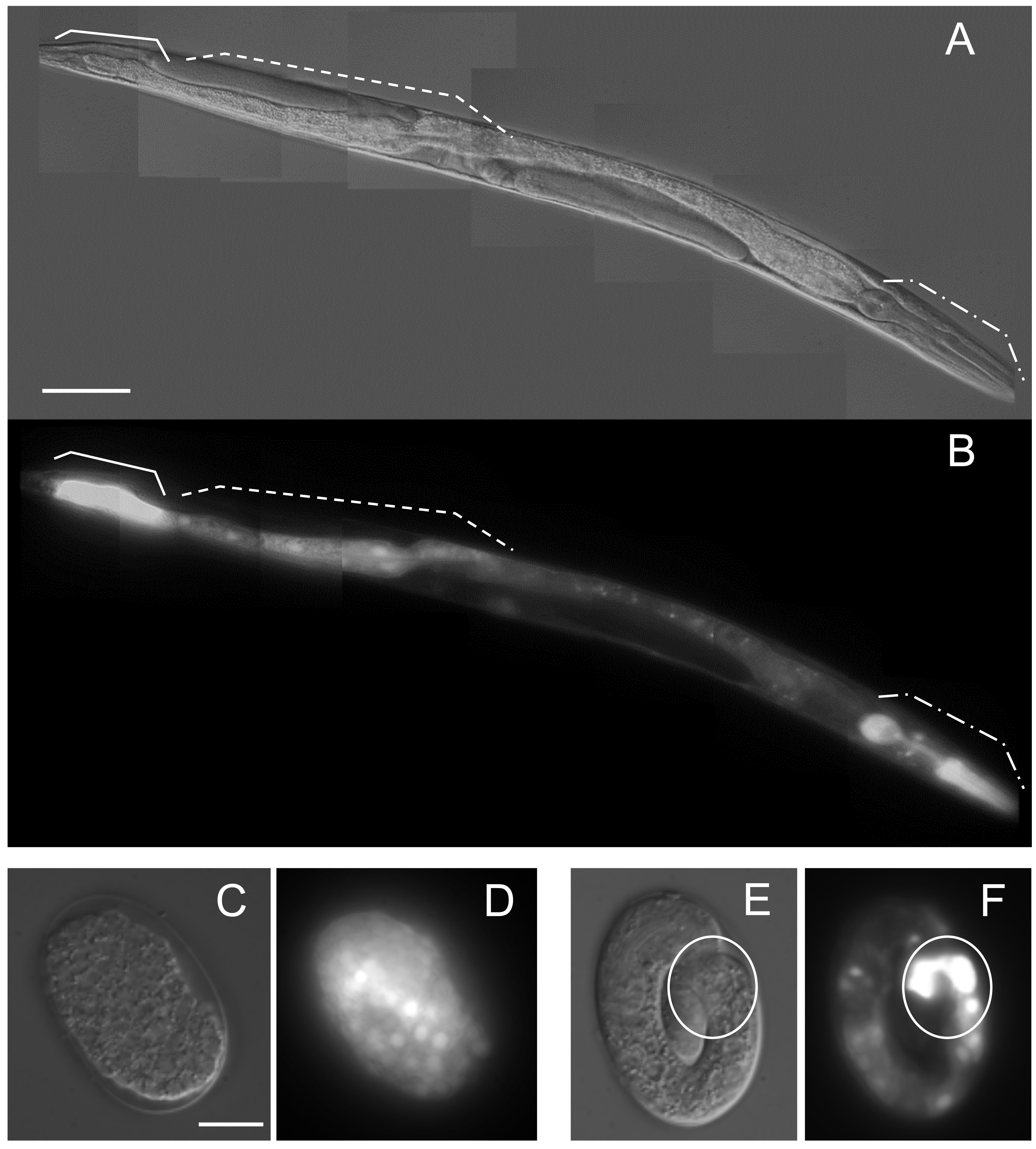

Supplement: Figure S13 — Expression of slr-2::GFP. DIC (A, C, E) and corresponding GFP (B, D, F) micrographs of an L4 larvae (A, B), a pre-morphogenetic embryo (∼300 cell stage; C, D), and late-stage embryo (E, F). Although brightest in the intestinal posterior, reporter expression was observed throughout the intestine in L4 stage larvae. In addition, L4 larvae exhibited expression in marginal cells and the m3VR, mC, I5, m5, m6, and m7 cells of the pharynx, as well as a small subset of head neurons and the excretory duct cell (A, B). Ubiquitous expression was observed in the early embryo (C, D), which becomes largely restricted to the intestine by the pretzel-stage, where expression is brightest in the posterior gut region (E, F). In panels A and B, the posterior region displaying the greatest intense intestinal fluorescence is delineated by a solid white brace and the adjacent dimmer region is delineated by a white, dashed brace. The foregut is delineated by a white dotted dashed brace. In panels E and F, the posterior intestinal region is circled. Scale bar: A, B 100 µm, C-F, 10 µm. (7.21 MB TIF) [file pgen.1000059.s013.tif]
