# Supplementary material for: Coordinated Regulation of Intestinal Functions in C. elegans by LIN-35/Rb and SLR-2
Source: PLoS Genet. 2008 Apr 25;4(4):e1000059. doi: 10.1371/journal.pgen.1000059 (PMC2312330; doi:10.1371/journal.pgen.1000059)
Supplement: Table S1 — Insulin and TOR signaling networks are down regulated in slr-2 mutants. (0.05 MB DOC) [file pgen.1000059.s014.doc]

**Table S1. Insulin and TOR signaling networks are down regulated in *slr-2* mutants.**

| ***H. sapiens* gene** | ***C. elegans* gene** | **Seq. name** | **Mean fold** |
| --- | --- | --- | --- |
| AAC | pod-2 | W09B6.1 | -1.8 |
| AMPK | aak-2 | T01C8.1 | -1.8 |
| CYS | gsy-1 | Y46G5A.31 | -1.5 |
| eIF4E | ife-3 | B0348.6 | -2.3 |
| eIF4E | ife-4 | C05D9.5 | -1.5 |
| ERK1/2 | mpk-1 | F43C1.2 | -2.1 |
| FOXO | daf-16 | R13H8.1 | -3.0 |
| GK |  | F14B4.2 | -1.7 |
| HIF1alfa | hif-1 | F38A6.3 | -1.8 |
| INSR | daf-2 | Y55D5A.5 | -1.6 |
| LAR | ptp-3 | C09D8.1 | -1.7 |
| MNK | mnk-1 | R166.5 | -1.7 |
| MO25 |  | Y53C12A.4 | -2.0 |
| mTOR | let-363 | B0261.2 | -2.4 |
| PDK1/2 | pdk-1 | H42K12.1 | -1.9 |
| PHK | cmd-1 | T21H3.3 | -1.9 |
| PKA | kin-1 | ZK909.2 | -1.5 |
| PP1 | gsp-1 | F29F11.6 | -3.0 |
| PP1 | gsp-2 | F56C9.1 | -1.6 |
| Raf | lin-45 | Y73B6A.5 | -2.2 |
| Ras | let-60 | ZK792.6 | -3.1 |
| Rheb |  | F54C8.5 | -3.1 |
| S6K1/2 | rskn-1 | T01H8.1 | -1.6 |
| SHC | shc-1 | F54A5.3 | -1.7 |
| TC10 | cdc-42 | R07G3.1 | -3.3 |

Human insulin and mTOR network members were obtained from the KEGG database (www.genome.jp/**kegg**/)
