# Supplementary material for: Coordinated Regulation of Intestinal Functions in C. elegans by LIN-35/Rb and SLR-2
Source: PLoS Genet. 2008 Apr 25;4(4):e1000059. doi: 10.1371/journal.pgen.1000059 (PMC2312330; doi:10.1371/journal.pgen.1000059)
Supplement: Table S2 — Description of tester genes used for qRT-PCR analysis. (0.05 MB DOC) [file pgen.1000059.s015.doc]

Table S2. Description of tester genes used for qRT-PCR analysis

| **Gene** | **Name** | **Category** | **Description** |
| --- | --- | --- | --- |
| C17C3.12 | *acdh-2* | metabolism/energy | a short-chain acyl-CoA dehydrogenase; plays a key role in energy production |
| C04F12.1 |  | metabolism/energy | translation initiation factor, general protein biosynthesis |
| C06E7.1 |  | metabolism/energy | S-adenosylmethionine synthase |
| ZK666.6 | *clec-60* | intestine-specific (1) | c-type lectin |
| ZK666.7 | *clec-61* | intestine-specific (3) | c-type lectin |
| C52E4.1 | *cpr-1* | intestine-specific (1,2,3) | cystein protease, is specifically expressed in the gut |
| F11A1.3 | *daf-12* | metabolism/energy | dauer formation pathway |
| Y55D5A.5 | *daf-2* | metabolism/energy | insulin receptor |
| C32H11.10 | *dod-21* | metabolism/energy | regulated by *daf-16* |
| H30A04.1 | *eat-20* | other | abnormal pharyngeal pumping |
| D2024.3 | *elo-3* | metabolism/energy | fatty acid elongase |
| C33D3.1 | *elt-2* | intestine-specific (1,4) | GATA-type transcription factor, is required redundantly with ELT-7 for initiating and maintaining terminal differentiation of the intestine |
| F21F8.4 |  | intestine-specific (1,2,3) | aspartyl protease |
| F57G4.5 |  | other | unnamed protein |
| F15B9.1 | *far-3* | metabolism/energy | fatty acid/retinol binding protein |
| F22B7.4 | *fip-1* | other | fungus induced protein |
| K08C7.5 | *fmo-2* | intestine-specific (1,2,3) | flavin-containing monoxygenase |
| T22G5.7 | *spp-12* | intestine-specific (1) | saposin-like protein |
| C03G6.13 | *tag-293* | other | secreted surface protein |
| ZK617.2 | *lips-6* | metabolism/energy | triacylglycerol lipase |
| ZK970.7 |  | other | unnamed protein |
| C36A4.8 | *brc-1* | cell cycle | a homolog of human BRCA1 |
| C37A2.4 | *cye-1* | cell cycle | G1 cell cycle regulator cyclin E |
| F02A9.6 | *glp-1* | germline | abnormal Germ Line Proliferation |
| C38C10.4 | *gpr-2* | cell cycle | GPR-2 appears to function redundantly during early embryogenesis and germ-line development to regulate chromosome and spindle movements during cell division |
| W03D2.4 | *pcn-1* | cell cycle | *C. elegans* ortholog of proliferating cell nuclear antigen (PCNA), an acidic protein that is an essential component of the DNA replication and repair machinery |
| C18G1.4 | *pgl-3* | germline | P-granule abnormality |
| F56A6.1 | *sago-2* | RNAi | an Argonaute homolog that is partially required for the amplification phase of RNAi responses |
| Y75B8A.22 | *tim-1* | cell cycle | DNA topoisomerase I-interacting protein |

(1) – gene from gut SAGE library (for criteria see Materials and Methods)

(2) – gene belongs to Mountain 8 (see Kim et al., 2001)

(3) – gene is expressed in the intestine only (NEXTDB database [http://nematode.lab.nig.ac.jp](http://nematode.lab.nig.ac.jp/) )

(4) – gene is expressed in the intestine only (Expression Patterns database <http://gfpweb.aecom.yu.edu/> )
